# Anti-Inflammatory Activity of the Major Triterpenic Acids of Chios Mastic Gum and Their Semi-Synthetic Analogues

**DOI:** 10.3390/biom14121618

**Published:** 2024-12-18

**Authors:** Panagiota Stamou, Despoina D. Gianniou, Ioannis P. Trougakos, Sofia Mitakou, Maria Halabalaki, Ioannis K. Kostakis, Alexios-Leandros Skaltsounis

**Affiliations:** 1 Department of Pharmacy, Division of Pharmaceutical Chemistry, National and Kapodistrian University of Athens, Panepistimiopolis Zografou, 15771 Athens, Greece; pstamou@pharm.uoa.gr; 2 Department of Cell Biology and Biophysics, Faculty of Biology, National and Kapodistrian University of Athens, 15784 Athens, Greece; gndespoina@biol.uoa.gr (D.D.G.); itrougakos@biol.uoa.gr (I.P.T.); 3 Department of Pharmacy, Division of Pharmacognosy and Natural Products Chemistry, National and Kapodistrian University of Athens, Panepistimiopolis Zografou, 15771 Athens, Greece; mitakou@pharm.uoa.gr (S.M.); mariahal@pharm.uoa.gr (M.H.)

**Keywords:** Chios Mastic Gum, triterpenic acids, 24*Z*-masticadienonic acid, 24*Z*-isomasticadienonic acid, anti-inflammatory activity, TNF-a, Il-6, NF-kB

## Abstract

24*Z*-Masticadienonic acid (MNA) and 24*Z*-isomasticadienonic acid (IMNA) are the major triterpenic acids in Chios Mastic Gum (CMG), a resin derived from *Pistacia lentiscus* var. *Chia*. Despite their promising pharmacological potential, limited information is available due to the complexity of isolating them in pure form. This study developed a chemo-selective method for isolating MNA and IMNA and investigated their chemical transformation through isomerization of the external double bond and A-ring contraction of the triterpene scaffold. A rapid method for isolating MNA from CMG was first established, followed by a high-yield acid-catalyzed procedure to obtain both 24*Z* and 24*E* isomers of IMNA. Additionally, a basic catalyzed isomerization of IMNA led to the formation of two new compounds with A-ring contraction, which could serve as novel scaffolds for the design of new triterpene analogs. The mixture of MNA/IMNA, along with the individual compounds and their semi-synthetic analogs, exhibited significant anti-inflammatory activity. Notably, 24*E*-isomasticadienonic acid and 24*Z*-2-hydroxy-3-oxotirucalla-1,8,24-trien-26-oic acid, a previously unreported compound, significantly reduced the mRNA expression levels of *Tnf*, *Il6*, and *Nfkb1* in RAW 264.7 macrophage cells.

## 1. Introduction

Inflammation constitutes a mechanism of protection from tissue damage or microbial invasion. Its purpose is to isolate and eliminate the harmful agents and damaged tissue while preparing the area for repair and healing. Although an inflammatory reaction is typically short-lived and results in the desired protective response, in some cases, it can be excessive or prolonged, causing significant tissue damage, organ dysfunction, and even death [1]. A key mediator in the inflammatory process is nuclear factor-κB (NF-κB), which is activated by pro-inflammatory cytokines such as tumor necrosis factor-α (TNF-α). Νuclear factor kappa-light-chain-enhancer of activated B cells (NF-κB) plays a pivotal role in the progression of inflammation [2]. In normal state, inhibitory κB (IκB) is bound to NF-κB [3]. Upon exposure to inflammatory stimuli, IκB kinase (IKK) phosphorylates IκB, leading to its degradation by the proteasome. This process allows NF-κB to be released and translocated from the cytoplasm to the nucleus, where it acts as a transcription factor promoting the expression of various cytokines and inflammatory mediators (e.g., iNOS, COX-2, TNF-α, IL-6, and IL-1β). Along with TNF-α and NF-κB, interleukin 6 (IL-6) is a classical pro-inflammatory cytokine produced through various immune responses that contribute to the initiation and progression of inflammation-related diseases. Therefore, regulating this kind of cytokines, and mediators is considered a complementary approach to managing inflammatory diseases in humans [4,5].

Chronic inflammation is a central contributor to the pathogenesis of numerous diseases, including cardiovascular disorders, neurodegenerative diseases, metabolic syndromes, and certain types of cancer [6]. The persistent activation of inflammatory pathways not only exacerbates these conditions but also drives their progression, highlighting the critical need for effective anti-inflammatory therapies [7]. Thus, targeting inflammation has emerged as a promising strategy to mitigate disease progression and improve health outcomes [8]. In recent years, an increasing number of studies have been conducted with the aim of exploiting natural products for the treatment of various diseases [9]. CMG is a notable example, historically recognized as a traditional remedy in the writings of Herodotus, Dioscurides, and Galen. Today, this aromatic resin has gained renewed interest due to its diverse biological properties, including anticancer, antioxidant, and anti-inflammatory activities [10].

CMG is derived from the evergreen shrub *Pistacia lentiscus* var. *Chia* of the Anacardiaceae family. Although *Pistacia lentiscus* is widespread across the Mediterranean basin, the resin is uniquely produced by mastic trees cultivated exclusively on the Greek island of Chios, particularly in its southern region [11]. The trees reach an age of 100 years, while resin production starts in their fifth year. The annual resin yield is estimated to range from 60 to 250 g per tree [12]. Since 1997, Chios masticha has been recognized as a Protected Designation of Origin (PDO) product by the European Union [13]. In 2015, the European Medicines Agency (EMA) issued a monograph supporting its use as a traditional herbal medicine for managing mild digestive disorders, addressing skin inflammations, and promoting wound healing [14]. Numerous studies further highlight the anti-inflammatory potential of CMG [15,16].

**Figure 1:**
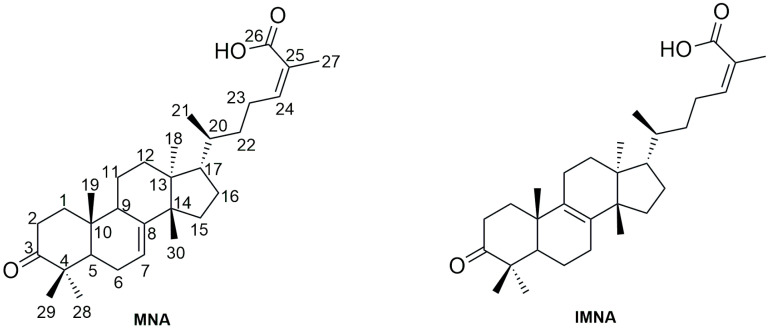
Chemical structures of the major triterpenic acids of CMG.

In CMG, approximately 120 constituents have been reported, classified into three groups: the polymer fraction (*cis*-1,4-poly-*β*-myrcene), the essential oil fraction, and terpenoids [6,7]. Terpenes constitute the major chemical group, accounting for about 65–70% of the total resin’s weight, and the majority belongs to triterpenoids. In more detail, approx. 25–27% comprises the neutral fraction (NF) and 38% the acidic fraction (AF). Notably, the characteristic triterpenic acids found in CMG are the 24*Z*-masticadienonic acid (MNA) and the 24*Z*-isomasticadienonic acid (IMNA), belonging to the group of tirucallane-type of triterpenes (Figure 1) [17,18]. It is important to note that the above-mentioned compounds have not been detected in other parts of the plant, such as the leaves [19]. The two isomers, MNA and IMNA, are found only in a limited number of plants, mainly in the *Pistacia* and *Amphipterygium* species, while they have been sporadically reported in *Schinus* and *Dysoxylum* species [20,21].

The biological activities of CMG are primarily attributed to its tetracyclic and pentacyclic triterpenic acids, such as oleanonic and moronic acids [22,23]. However, limited data exists regarding the biological profiles of its major triterpenic acids, MNA and IMNA [24,25]. This gap in knowledge mainly arises from the challenges associated with isolating these isomers in their pure form, as they differ only in the position of the double bond, C-7/C-8 for MNA and C-8/C-9 for IMNA.

To address these challenges and explore the significant potential of MNA and IMNA, this study aimed to develop methods for their isolation using advanced chromatographic techniques or chemical reactions. Following isolation, their anti-inflammatory activity was evaluated. Additionally, the synthesis of MNA and IMNA analogs was pursued to investigate structure–activity relationships and potentially identify more potent derivatives. The discovery of new compounds within this triterpenic framework could be of significant interest, considering the limited data currently available on this chemical core.

## 2. Materials and Methods

### 2.1. Materials

Ethyl acetate (EtOAc), *n*-Hexane, methanol (MeOH), dichloromethane (DCM), and cyclohexane used for extraction and purification were of analytical grade (Fisher Scientific, Loughborough, Belgium), while water (H_2_O) was distilled. For the pH adjustment, sodium hydroxide pellets (NaOH- penta CHEMICALS UNITED, Prague, Czech Republic) and hydrochloric acid (HCl- analytical grade; Fisher Scientific, Loughborough, Belgium) were used. Acetonitrile (ACN, Avantor Performance Materials, Poland) and H_2_O (Fisher Scientific, Loughborough, Belgium) used for preparative High-Performance Liquid Chromatography (prep-HPLC) analysis were of HPLC grade. The reactions were monitored by analytical Thin-Layer Chromatography (TLC) and the compounds were visualized on 254 and 366 nm (UV lamp). Flash chromatography was performed on Merck silica gel 60A (0.040–0.060 mm) (Merck, Kenilworth, NJ, USA). All commercially available reagents were purchased from Alfa Aesar (Ward Hill, MA, USA) and used without any further purification. Optical rotations were measured with a Jasco P-2000 (Tokyo, Japan) polarimeter. ^1^H NMR, ^13^C NMR, and 2D spectra were recorded on a Bruker Avance III 600 MHz spectrometer (Bruker Biospin AG, Faellanden, Switzerland) and on a Bruker Avance NEO 400 MHz spectrometer (Bruker Biospin AG, Faellanden, Switzerland) in deuterated Chloroform-D1 (CDCl_3_) and Methanol-D4 (MeOD) (Sigma-Aldrich, St. Louis, MO, USA) (see Supplementary Materials). Chemical shifts are expressed as δ values in parts per million (ppm), and the coupling constants (*J*) are given in Hertz (Hz). The signals of ^1^H and ^13^C NMR spectra were unambiguously assigned by using 2D NMR techniques: ^1^H-^1^H COSY, HSQC, NOESY, and HMBC experiments. The signals are reported as follows: (s = singlet, d = doublet, t = triplet, m = multiplet, br = broad). Topspin software version 4.2.0 (Bruker Biospin AG, Faellanden, Switzerland) was used for the acquisition and processing of the spectra. The samples were analyzed using the following analytical platforms: a UPLC combined with a hybrid time of flight (triple-TOF 5600+, AB SCIEX) system and a UPLC coupled with a Velos Pro-IT-Orbitrap ELITE MS (Thermo Scientific) analyzer. Data acquisition was performed using Analyst 1.7.1 software (AB Sciex, Framingham, MA, USA) for the former and Thermo Xcalibur 2.2.44 software (Thermo Fischer Scientific, Waltham, MA, USA) for the latter. In both platforms, LC analysis was performed on an ACQUITY H-Class UPLC system (Waters, Wilimslow, UK) equipped with a binary solvent manager and an FTN sample manager. All separations were carried out using reversed-phase chromatography columns. For the triple-TOF, a Fortis Speedcore Biphenyl (2.6 μm, 2.1 mm × 100 mm) was used at a stable temperature of 40 °C. The solvent system consisted of (A) H_2_O containing 0.1% formic acid and (B) ACN. The gradient elution program started with 5% of B, which was held for 2 min, and in the next 15 min, B reached 100% and was held for 2 min. Finally, the system returned to the initial conditions for system equilibration. Measurements were performed with a total acquisition time of 20 min and a flow rate of 300 μL/min. The injection volume was 10 μL, and the autosampler temperature was set to 10 °C. For the Orbitrap instrumentation, separations were performed on a Supelco Ascentis. All the parameters used were the same, but there were some differences in the elution gradient. The gradient elution program started with 5% of B, which was held for 1 min, and in the next 14 min, B reached 100% and was held for 2 min. Finally, the system returned to the initial conditions for system equilibration. Detection was employed using negative electrospray ionization (ESI) modes for each instrumentation. The UPLC-triple-TOF platform was equipped with a DuoSpray ion source, while for the Orbitrap a heated ESI ion source was utilized. For the triple-TOF, the data-dependent acquisition mode covered a mass range of 100–1000 Da with a source temperature of 450 °C and a voltage set at −4500 V. The Duospray source and the exhaust gas pressure were maintained at 40 psi, while the curtain gas pressure was set at 35 psi. In the Orbitrap system, detection was performed with a source temperature of 350 °C, with voltage settings of −2700 V. The sheath gas flow rate was maintained at 45 μL/min, while the auxiliary flow rate was set to 15 μL/min. The resolution of the Orbitrap platform was adjusted to 60,000 for precursor ion spectrums. The HRMS data were acquired in full scan mode in the range of 113–1000 *m*/*z*, with a resolving power of 60,000 at 500 *m*/*z* and a scan rate of 1 microscan per second.

### 2.2. CMG Fractionation and Isolation

#### 2.2.1. Polymer Removal Procedure

CMG was kindly provided by the Chios Mastiha Growers Association (CMGA). Firstly, a decantation process as described by Paraschos et al. was followed, resulting in the Total Mastic Extract Without Polymer (TMEWP) [26]. Briefly, 500.0 g of CMG were dissolved in 500.0 mL of EtOAc, and then 1.5 L of MeOH was added. Allowing the mixture to stand for 2 days, a layer of *cis*-poly-*β*-myrcene (150.0 g) was separated. After the filtration and evaporation of the solution, 350.0 g of TMEWP were obtained as a white powder (Figure S1).

#### 2.2.2. Pilot Extraction of the NF and AF of Triterpenes of CMG

A total of 350.0 g of TMEWP was diluted in the organic phase consisting of EtOAC and *n*-Hexane (2:8 *v*/*v*, 3.0 L). Next, 20% NaOH was added in the aqueous phase, consisting of MeOH and H_2_O (1:1 *v*/*v*, 3.4 L), until reaching a pH of 12. TMEWP partitioned between the aqueous and organic phases. Triterpenic acids were found as sodium salts in the aqueous phase, while the NF of triterpenes dispersed into the organic phase. Following solvent evaporation, the non-polar extract afforded a total of 170.0 g of mastic’s NF. The aqueous phase was then acidified with a solution of 6N HCl until pH = 3. This acidic solution was extracted three consecutive times with DCM, each time using 1.0 L. The resulting organic phase contains the AF of TMEWP, which, once evaporated, afforded 180.0 g. All solvent evaporations were performed using a rotary evaporator (Buchi) with a water bath at 40 °C (Figure S1).

#### 2.2.3. Isolation of the Major Triterpenic Acids

The isolation of MNA and IMNA was performed using preparative RP-HPLC (Buchi, Pure C-850 FlashPrep) hyphenated to a Photo Diode Array (PDA) detector. In more detail, 500.0 mg of the AF were injected in a reversed-phase column (Agilent 5 Prep-C18, 50 × 50 mm). Elution was carried out with a solvent system consisting of H_2_O (A) and ACN (B) using gradient elution with a flow rate of 100.0 mL/min, which afforded 50.0 mg of pure MNA and 200.0 mg of an MNA/IMNA mixture 45:55, respectively. The elution method started at 80% B, which was maintained for 3 min and increased to 90% in 2 min, which was maintained for 6.5 min. Then, the percentage of B increased to 100% in 1.5 min and was maintained for 2 min, before returning to the initial conditions in 1 min for a 1 min re-equilibration (17 min in total). UV detection was performed at a wavelength of 210 nm. The structure of isolated MNA was confirmed by LC-ESI-HRMS and 1D and 2D NMR data.

24*Z*-masticadienonic acid (MNA): [${a]}_{25}^{589}$ = −28 (c 0.196, MeOH).^1^H NMR (400 MHz, CDCl_3_) δ 6.07 (t, *J* = 7.5 Hz, 1H, H-24), 5.30 (m, 1H, H-7), 2.75 (td, *J* = 5.4/14.5 Hz, 1H, H-2a), 2.51 (m, 2H, H-23), 2.28 (m, 1H, H-9), 2.24 (m, 1H, H-2b), 2.12–2.06 (m, 2H, H-6), 1.99 (m, 1H, H-1a), 1.94 (m, 1H, H-11a), 1.92 (s, 3H, CH_3–_27), 1.80 (m, 1H, H-16a), 1.72 (m, 1H, H-5), 1.65 (m, 1H, H-16b), 1.59 (m, 1H, H-15a), 1.51 (m, 1H, H-15b), 1.53 (m, 1H, H-22a), 1.52–1.42 (m, 2H, H-12), 1.49 (m, 1H, H-17), 1.46 (m, 1H, H-1b), 1.40 (m, 1H, H-20), 1.25 (m, 1H, H-11b), 1.13 (m, 1H, H-22b), 1.11 (s, 3H, CH_3_-29), 1.04 (s, 3H, CH_3_-28), 1.01 (s, 3H, CH_3_-30), 1.00 (s, 3H, CH_3_-19), 0.89 (d, *J* = 6.3, 3H, CH_3_-21), 0.80 (s, 3H, CH_3_-18). ^13^C NMR (100 MHz, CDCl_3_) δ 217.1 (C, C-3), 172.5 (C, C-26), 147.3 (CH, C-24), 146.1 (C, C-8), 125.9 (C, C-25), 118.0 (CH, C-7), 53.1 (CH, CH-17), 52.5 (CH, C-5), 51.4 (C, C-14), 48.7 (CH, C-9), 48.0 (C, C-4), 43.7 (C, C-13), 38.7 (CH_2_, C-1), 36.2 (CH, C-20), 35.8 (CH_2_, C-22), 35.2 (C, C-10), 35.1 (CH_2_, C-2), 34.2 (CH_2_, C-12), 33.8 (CH_2_, C-16), 28.4 (CH_2_, C-11), 27.6 (CH_3_, C-30), 27.1 (CH_2_, C-23), 24.7, 24.5 (CH_2_, C-6), 22.1 (CH_3_, C-18), 21.8, 20.7 (CH_3_, C-27), 18.5 (CH_3_, C-21), 18.4 (CH_2_, C-15), 13.0 (CH_3_, C-19). ESΙ, *m*/*z* [M-H]^−^ calcd. for C_30_H_45_O_3_^−^: 453.3374, found: 453.3365.

### 2.3. Acid-Catalyzed Synthesis of IMNA

#### 2.3.1. Synthesis of IMNA from MNA

To a solution of MNA (60.0 mg, 0.13 mmol) in dry DCM (23.0 mL) at 0 °C, under argon, 1.0 eq of boron tribromide (BBr_3_) was added. The resulting mixture was stirred at 0 °C for 15 min. After completion of the reaction, the residue was dissolved in DCM, washed with H_2_O, dried over anhydrous sodium sulfate (Na_2_SO_4_), filtrated, and vacuum evaporated. After workup, 50.0 mg of IMNA was obtained as a white powder. Yield: 83%. [${a]}_{25}^{589}$ = +23 (c 0.355, MeOH). ^1^H NMR (600 MHz, CDCl_3_) δ 6.08 (td, *J* = 1.3/7.5 Hz, 1H, H-24), 2.62 (m, 1H, H-23a), 2.54 (m, 1H, H-2a), 2.45 (m, 1H, H-2b), 2.38 (m, 1H, H-23b), 2.14 (m, 1H, H-7a), 2.09 (m, 1H, H-7b), 1.99 (m, 1H, H-1a), 1.95 (m, 1H, H-16a), 1.91 (br, 3H, CH_3_-27), 1.88 (m, 1H, H-16b), 1.71 (m, 2H, H-11) 1.69 (m, 1H, H-5), 1.67 (m, 2H, H-12), 1.62 (m, 1H, H-1b), 1.61 (m, 1H, H-6a), 1.53 (m, 1H, H-15a), 1.48 (m, 1H, H-6b), 1.43 (m, 1H, H-20), 1.25 (m, 1H, H-15b), 1.12 (m, 2H H-22), 1.09 (s, 3H, CH_3_-29), 1.04 (s, 3H, CH_3_-28), 1.04 (s, 3H, CH_3_-19), 0.92 (d, *J* = 6.25 Hz, 3H, CH_3_-21), 0.88 (s, 3H, CH_3_-30), 0.75 (s, 3H, CH_3_-18). ^13^C NMR (151 MHz, CDCl_3_) δ 218.3 (C, C-3), 173.4 (C, C-26), 147.4 (CH, C-24), 134.8 (C, C-8), 132.8 (C, C-9), 125.9 (C, C-25), 51.6 (CH, C-5), 50.2 (C, C-13), 50.1 (CH, C-17), 47.4 (C, C-4), 44.2 (C, C-14), 37.2 (C, C-10), 36.5 (CH, C-20), 35.9 (CH_2_, C-22), 35.6 (CH_2_, C-1), 34.6 (CH_2_, C-2), 30.8 (CH_2_, C-12), 29.9 (CH_2_, C-15), 28.1 (CH_2_, C-11), 27.6 (CH_2_, C-7), 26.9 (CH_2_, C-23), 26.8 (CH_3_, C-29), 24.3 (CH_3_, C-30), 21.4 (CH_2_, C-16), 21.2 (CH_3_, C-28), 20.6 (CH_3_, C-27), 20.4 (CH_2_, C-6), 19.9 (CH_3_, C-19), 18.7 (CH_3_, C-21), 15.6 (CH_3_, C-18). ESΙ *m*/*z* [M-H]^−^ calcd. for C_30_H_45_O_3_^−^: 453.3374, found: 453.3411.

#### 2.3.2. Synthesis of 24*E*-Isomasticadienonic Acid (**1**) from MNA

To a solution of MNA (60.0 mg, 0.13 mmol) in dry DCM (23.0 mL) at 0 °C, under argon, 1.2 eq of BBr_3_, were added. The resulting mixture was stirred at 0 °C for 40 min. After completion of the reaction, the residue was dissolved in DCM, washed with H_2_O, dried over anhydrous Na_2_SO_4_, filtrated, and concentrated under reduced pressure. The crude product was purified by flash chromatography (silica gel), using cyclohexane/EtOAc, 100→88:12 *v*/*v* as the eluent, to afford 20.0 mg of 1 as a white powder. Yield: 35%, [${a]}_{25}^{589}$ = −7.0 (c 0.298, MeOH). ^1^H NMR (600 MHz, CDCl_3_) δ 6.90 (td, 1H, *J* = 1.3/7.1 Hz, H-24), 2.54–2.45 (m, 2H, H-2), 2.31 (m, 1H, H-23a), 2.14 (m, 1H, H-2a), 2.09 (m, 1 H, H-2b), 2.04 (m, 1H, H-23b), 1.99 (m, 1H, H-1a), 1.95 (m, 1H, H-16a), 1.88 (m, 1H, H-16b),1.84 (br, 3H, CH_3_-27), 1.71 (m, 2H, H-11) 1.69 (m, 1H, H-5), 1.67 (m, 2H, H-12), 1.62 (m, 1H, H-1b), 1.61 (m, 1H, H-6a), 1.53 (m, 1H, H-15a), 1.48 (m, 1H, H-6b), 1.43 (m, 1H, H-20), 1.25 (m, 1H, H-15b), 1.13 (m, 2H, H-22), 1.09 (s, 3H, CH_3_-29), 1.05 (s, 3H, CH_3_-28), 1.04 (s, 3H, CH_3_-19), 0.94 (d, *J* = 6.1 Hz, 3H, CH_3_-21), 0.89 (s, 3H, CH_3_-30), 0.75 (s, 3H, CH_3_-18). ^13^C NMR (151 MHz, CDCl_3_) δ 218.4 (C, C-3), 172.9 (C, C-26), 145.9 (CH, C-24), 134.8 (C, C-8), 132.9 (C, C-9), 126.7 (C, C-25), 51.6 (CH, C-5), 50.3 (C, C-13), 50.2 (CH, C-17), 47.4 (C, C-4), 44.3 (C, C-14), 37.3 (C, C-10), 36.5 (CH, C-20), 35.7 (CH_2_, C-1), 35.0 (CH_2_, C-22), 34.7 (CH_2_, C-2), 30.9 (CH_2_, C-12), 29.9 (CH_2_, C-15), 28.2 (CH_2_, C-11), 27.6 (C-7), 26.9 (CH_3_, C-29), 26.1 (CH_2_, C-23), 24.3 (CH_3_, C-30), 21.5 (CH_2_, C-16), 21.3 (CH_3_, C-28), 20.4 (CH_2_, C-6), 19.9 (CH_3_, C-19), 18.7 (CH_3_, C-21), 15.7 (CH_3_, C-18), 12.1 (CH_3_, C-27). ESI, *m*/*z* [M-H]^−^ calcd. for C_30_H_43_O_3_^−^: 453.3374, found: 453.3395.

#### 2.3.3. Synthesis of IMNA from MNA/IMNA Mixture

**Figure 2:**
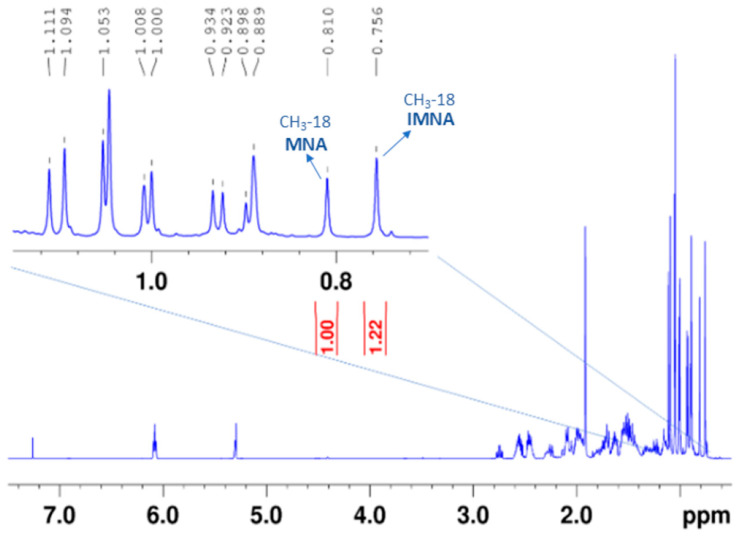
^1^H NMR spectrum of MNA/IMNA mixture (600 MHz, CDCl_3_). In the zoomed region, the characteristic peaks of the MNA/IMNA methyl groups are highlighted.

To a solution of MNA/IMNA mixture (200.0 mg, 45:55) in dry DCM (76.0 mL) at 0 °C, under argon, 1.0 eq of boron tribromide (BBr_3_) as per MNA was added. The equivalents of BBr_3_ were calculated based on the quantity of MNA on the ^1^H NMR spectra of the mixture (Figure 2). The resulting mixture was stirred at 0 °C for 15 min. After completion of the reaction, the residue was dissolved in DCM, washed with H_2_O, dried over anhydrous sodium sulfate (Na_2_SO_4_), filtrated, and vacuum evaporated. After workup, 160.0 mg of IMNA was obtained as a white powder (yield: 83%).

### 2.4. Basic Catalyzed Synthesis of IMNA Analogs

To a solution of IMNA (0.12 mmol) in *n*-Heptane:DMSO (5:1 *v*/*v*, 7.32:1.46 mL), 8 eq of potassium tert-butoxide (t-BuOK) were added. After stirring at room temperature for 40 min under argon atmosphere, the resulting mixture was acidified with 1N HCl solution. The mixture was then extracted with EtOAc, washed with a saturated sodium bicarbonate solution, H_2_O, and a brine solution, dried over anhydrous Na_2_SO_4_, and concentrated to dryness. The crude product was purified by flash chromatography on silica gel, using DCM (A)/MeOH (B) as the eluent (starting at 100% A and gradually decreasing to 80% A, with 20% B), resulting in the purification of 24*Z*-2-hydroxy-3-oxotirucalla-1,8,24-trien-26-oic acid (2) and 24*Z*-3b-hydroxy-1(2→3)-abeotirucalla-8,24-dien-2,26-dioic acid (3).

Physicochemical data of analog **2**: Yield: 16%, [${a]}_{25}^{589}$ = −8 (c 0.332, MeOH). ^1^H NMR (600 MHz, CDCl_3_, ppm) δ 6.56 (s,1H, H-1), 6.08 (t, *J* = 7.3 Hz, 1H, H-24), 2.56 (m, 1H, H-23a), 2.45 (m, 1H, H-23b), 2.18 (m, 1H, H-7a), 2.16 (m, 1H, H-12a), 2.04 (m, 1H, H-12b), 2.02 (m, 1H, H-7b), 2.02 (m, 1H, H-5), 1.91 (s, 3H, CH_3_-27), 1.73 (m, 1H, H-11a), 1.67 (m, 2H, H-6), 1.65 (m, 1H, H-11b), 1.54 (m, 2H, H-15), 1.52 (m, 1H, H-22a), 1.51 (m, 1H, H-17), 1.42 (m, 1H, H-20), 1.41 (m, 1H, H-22b), 1.22 (s, 3H, H-28), 1.21 (s, 1H, H-19), 1.10 (s, 3H, CH_3_-29), 0.93 (d, *J* = 6.29 Hz, 3H, CH_3_-21), 0.91 (s, 3H, CH_3_-30), 0.74 (s, 3H, CH_3_-18). ^13^C NMR (151 MHz, CDCl_3_) δ 201.2 (C, C-3), 173.0 (C, C-26), 147.4 (CH, C-24), 144.2 (C, C-2), 135.0 (C, C-8), 131.5 (C, C-9), 125.9 (CH, C-1), 125.9 (C, C-25), 50.4 (C, C-13), 50.1 (CH, C-17), 48.3 (CH, C-5), 44.2 (C, C-14), 43.9 (C, C-4), 39.2 (C, C-10), 36.6 (CH, C-20), 36.0 (CH_2_, C-22), 30.9 (CH_2_, C-11), 29.9 (CH_2_, C-15), 28.2 (C, C-10), 27.0 (CH_2_, C-23), 26.4 (CH_2_, C-12), 26.3 (CH_3_, C-28), 25.4 (CH_3_, C-19), 24.8 (CH_3_, C-30), 23.3 (CH_2_, C-7), 21.7 (CH_3_, C-29), 20.7 (CH_3_, C-27), 19.2 (CH_2_, C-6), 18.8 (CH_3_, C-21), 15.7 (CH_3_, C-18). ESI, *m*/*z* [M-H]^−^ calcd. for C_30_H_43_O_4_^−^: 467.3167, found: 467.3161.

Physicochemical data of analog **3**: Yield: 48%, [${a]}_{25}^{589}$ = −28 (c 0.321, MeOH). ^1^H NMR (600 MHz, MeOD) δ 5.95 (td, *J* = 1.3/7.5 Hz, 1H, H-24), 2.48 (m, 2H, H-23), 2.41 (m, 1H, H-1a), 2.11 (m, 2H, H-11), 2.10 (m, 2H, H-7), 1.87 (s, 3H, CH_3_-27), 1.76 (m, 2H, H-12), 1.71 (m, 1H, H-1b), 1.67 (m, 1H, H-5), 1.61 (m, 1H, H-15a), 1.57 (m, 2H, H-6), 1.54 (m, 1H, H-17), 1.54 (m, 1H, H-22a), 1.45 (m, 1H, H-20), 1.34 (m, 2H, H-16), 1,22 (m, 1H, H-15b), 1.16 (m, 1H, H-22b), 1.14 (s, 3H, CH_3_-19), 0.99 (s, 3H, CH_3_-28,), 0.99 (s, 3H, CH_3_-29), 0.95 (d, *J* = 6.4 Hz, 3H, CH_3_-21), 0.91 (s, 3H, CH_3_-30), 0.82 (s, 3H, CH_3_-18). ^13^C NMR (151 MHz, MeOD) δ 179.4 (C, C-3), 171.6 (C, C-26), 144.2 (CH, C-24), 136.9 (C, C-9), 134.3 (C, C-8), 128.4 (C, C-25), 88.4 (C, C-2), 58.7 (CH, C-5), 51.4 (CH, C-17), 50.5 (C, C-13), 48.5 (CH2, C-1), 47.7 (C, C-4), 45.8 (C, C-14), 44.7 (C, C-10), 37.6 (CH, C-20), 37.0 (CH_2_, C-22), 31.8 (CH_2_, C-12), 31.0 (CH_2_, C-15), 29.0 (CH_2_, C-16), 28.1 (CH_2_, C-7), 28.0 (C-29), 27.5 (CH_2_, C-23), 24.7 (CH_3_, C-30), 23.5 (CH_2_, C-11), 21.5 (CH_3_, C-19), 21.0 (CH_3_, C-27), 21.0 (CH_3_, C-28), 19.8 (CH_2_, C-6), 19.1 (CH_3_, C-21), 15.9 (CH_3_, C-18). ESI, *m*/*z* [M-H]^−^ calcd. for C_30_H_45_O_5_^−^: 485.3275, found: 485.3307.

### 2.5. Biological Assays

#### 2.5.1. Cell Line and Cell Culture Conditions

Murine macrophage RAW 264.7 cells were purchased from the American Tissue Culture Collection (ATCC). Cells were cultured in Dulbecco’s modified Eagle’s medium (ThermoFisher Scientific Inc., Waltham, MA, USA), supplemented with 10% (*v*/*v*) fetal bovine serum and 2 mM glutamine in a humidified incubator at 5% CO_2_ and 37 °C. Cell treatment with compounds and/or LPS (1 µg/mL). was performed for 24 h.

#### 2.5.2. Cell Survival Assay

To determine the cytotoxic effect, cells were seeded onto 96-well plates (Corning, Inc., New York, NY, USA) at a density of 3000 cells/well and treated with various concentrations of compounds and/or LPS (1 µg/mL). Following incubation for 24 h, the medium was replaced by 3-(4,5-dimethylthiazol-2-yl)-2,5-diphenyltetrazolium bromide (M5655, Sigma-Aldrich, Taufkirchen, Germany) dissolved at a final concentration of 1 mg/mL in serum-free, phenol red-free medium. The formed formazan crystals were then dissolved by isopropanol, and the absorbance of the solution was measured at a 570 nm wavelength.

#### 2.5.3. RNA Extraction, cDNA Synthesis and Quantitative Real-Time PCR (Q-RT-PCR) Analysis

Total RNA was extracted from cells using RNAiso plus (Takara Bio Inc., Shiga, Japan) and quantified with a BioSpec-nano spectrophotometer (Shimadzu Inc., Kyoto, Japan). Subsequently, 1 μg RNA was converted to cDNA using the FastGene Scriptase II cDNA Kit (NIPPON Genetics, Düren, Germany). To carry out Quantitative Real-Time PCR analyses, the 5x HOT FIREPol^®^ EvaGreen^®^ qPCR Supermix (Solis BioDyne, Tartu, Estonia) and PikoRealTM Real-Time PCR System (Thermo Fisher Scientific Inc., Waltham, MA, USA) were used. Primers were designed using the primer-BLAST tool (http://www.ncbi.nlm.nih.gov/tools/primer-blast/, accessed on 12 February 2019).

## 3. Results

The first step of the current study aimed to establish a simple and straightforward procedure for isolating MNA and IMNA, the major compounds of the acidic fraction of CMG. As previously noted, the removal of *cis*-1,4-poly-*β*-myrcene was essential to simplify the isolation process. TMEWP was obtained by following the decantation method described by Paraschos et al. [26]. Then, subsequent liquid–liquid extractions were conducted on a large scale using gradual pH adjustments (basic to acidic) to separate neutral and acidic triterpenes. From 350.0 g of TMEWP, the NF was isolated using an *n*-Hexane:EtOAc:MeOH mixture (6:4:1:1), with NaOH for aqueous alkalization. Acidification of the aqueous phase with HCl, followed by DCM extraction, yielded the AF containing MNA, IMNA, oleanonic acid, and moronic acid, as confirmed by RP-HPLC analysis (Figure S2). For purification, a preparative RP-HPLC-PDA method was developed. Each 17 min injection of 500.0 mg of AF yielded 50.0 mg of pure MNA and 200.0 mg of an MNA/IMNA mixture in a 40:60 ratio (Figure S3).

**Scheme 1:**
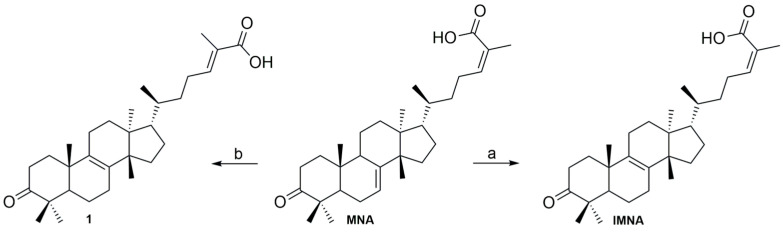
Synthesis of IMNA and **1**. a. BBr_3_, DCM, 15 min, 83%; b. BBr_3_, DCM, 40 min, 35%.

Although pure MNA was isolated in small quantities, its transformation to IMNA through chemo-selective double bond migration was explored [27,28,29,30,31]. The presence of two double bonds, one internal and one external, in the chemical structure of MNA presents a significant synthetic challenge for achieving selective migration. Thus, extra caution is necessary to prevent the scrambling of the internal bond migration and the possible *E*/*Z* isomerization of the external double bond. The existing literature confirms that double bond isomerization can occur under both acidic and basic conditions [32,33]. Furthermore, it is well-established that in olefin double bond isomerization, the *E*-configuration is predominantly favored due to thermodynamic preference [34]. Consequently, the investigation focused on the effects of various Lewis acids (CF_3_CO_2_H and BF_3_^.^Et_2_O) on MNA, but no double bond migration was observed, as monitored by NMR analysis. In contrast, treatment of MNA with BBr_3_, a relatively strong Lewis acid, facilitated the desired double bond migration, enabling the synthesis of IMNA. Numerous experiments revealed that the quantity of acid equivalents and the reaction time play a significant role. Intriguingly, the addition of 1.0 eq of BBr_3_ for 15 min affords IMNA in 83% yield. Extending the reaction to 1.2 equivalents for 40 min produced another IMNA isomer in a 35% yield, as confirmed by LC-ESI-HRMS (C_30_H_46_O_3_) (Scheme 1).

These two isomers could be distinguished based on the configuration of the external double bond, located at position C-24/C-25, indicating the existence of two isomers, *Z* and *E*. The structure of these derivatives was further validated using 1D (^1^H and ^13^C) and 2D (COSY, HSQC, HMBC, and NOESY) NMR experiments. Specifically, H-24 of the *Z*-isomer (IMNA) resonates at 6.08 ppm, whereas in 1, it resonates at 6.89 ppm. The confirmation of the structural assignment for the two isomers was solidified through the NOESY experiment, where H-24 of 24*Z*-IMNA exhibited NOEs with the protons of CH_3_-27, whereas this correlation was absent for **1** (Figure S17). In the ^13^C ΝMR spectrum of these two compounds, the key difference between them is observed in relation to CH_3_-27. In IMNA, CH_3_-27 resonates at 20.6 ppm, while in analog 1 at 12.13 ppm (Figures S14 and S21).

Building on the established method for double bond translocation and isomerization, we extended its application to the MNA/IMNA mixture obtained from the preparative RP-HPLC method. Given that this mixture represents the major fraction from the chromatographic process, optimizing this transformation directly from this source offers significant advantages. Remarkably, the procedure proved successful, enabling the selective conversion of the MNA/IMNA mixture to pure IMNA, or to its isomer, on a gram scale. In this case, the equivalents of BBr_3_ were calculated based on the moles of MNA present in the mixture. To quantify MNA, the integration of the ^1^H NMR peaks corresponding to CH_3_-18 of MNA was compared with the analogous methyl group signal of IMNA (Figure 2). This streamlined approach, which eliminates the need for the initial separation of MNA, highlights the efficiency and versatility of the developed method, offering significant potential for larger-scale applications.

Following the successful production of pure IMNA from the mixture of the isomers in significant quantities, and since double bond isomerization can also occur under basic conditions, the base-catalyzed double bond migration was investigated. For the initial experiments, potassium tert-butoxide (*t*-BuOK) was selected due to its strong basicity, while the MNA/IMNA mixture was used to replicate previous successes. However, these preliminary attempts resulted in complex mixtures that were difficult to purify and analyze. Encouragingly, using 4.0 equivalents of *t*-BuOK in an *n*-Heptane:DMSO mixture led to the detection of a new compound, as indicated by LC-ESI-HRMS data. Specifically, an ion was detected at *m*/*z* 467.3157 in negative ion mode, with a proposed molecular formula of C_30_H_44_O_4_, potentially corresponding to an oxidized derivative of the starting compound. Although the reaction did not yield the desired MNA product, we decided to continue working with this method, as every new isomer of this scaffold could be valuable.

**Scheme 2:**
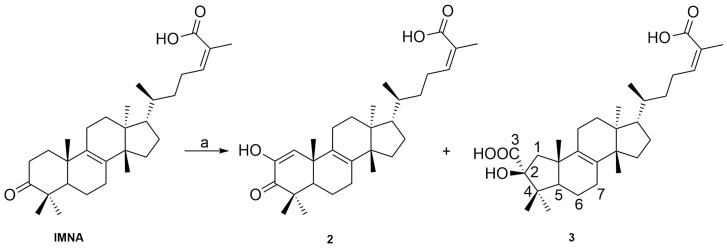
Synthesis of **2** and **3**. a. *t*-BuOK, *n*-Heptane:DMSO.

To streamline the procedure, slight adjustments were made, and the reaction was conducted using pure 24*Z*-IMNA. Specifically, the addition of 8.0 equivalents of *t*-BuOK to a pure solution of IMNA in *n*-Heptane:DMSO led to the synthesis of compounds **2** and **3** (Scheme 2). Interestingly, rather than facilitating the double bond migration, the treatment of IMNA with *t*-BuOK resulted in the formation of two new analogs with A-ring contraction of the triterpene moiety.

**Scheme 3:**
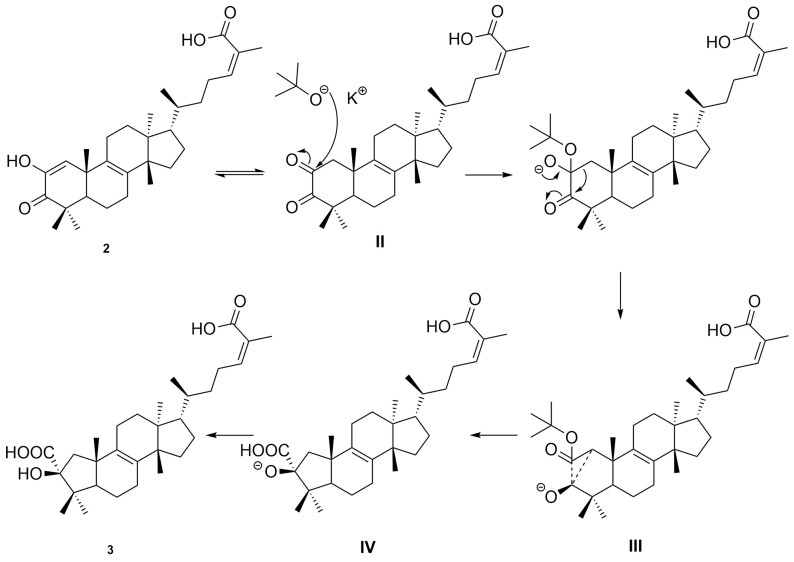
Plausible mechanism of the formation of compound **3**.

Under these conditions, oxidation occurred initially, followed by benzilic acid rearrangement and the formation of compound **3**. The plausible mechanism of the synthesis of compound **3** is depicted in Scheme 3. Initially, oxidation takes place, resulting in the formation of the *α*-hydroxy analog **2**, which exists in equilibrium with the diketone form II. Subsequently, the *tert*-butoxide anion attacks the less obstructed ketone group through a nucleophilic addition, forming the alkoxide III. Consequently, through a bond rotation, the methylene group attacks the *α*-carbonyl group, leading to the formation of the *α*-hydroxy–carboxylic acid **3**. The structures of **2** and **3** were unambiguously determined through ^1^H and ^13^C NMR spectra, using both direct and long-range heteronuclear correlation experiments (HMBC and HMQC) and LC-ESI-HRMS spectra.

As for the structural elucidation of analog **2**, in the LC-ESI-HRMS spectra, an ion was detected at *m*/*z* 467.3162 [M–H]^−^, with a proposed molecular formula of C_30_H_44_O_4_, corresponding to an oxidized version of IMNA featuring an additional oxygen atom. In the ^13^C spectrum, two new peaks were observed at 144.2 ppm and 125.9 ppm, indicating the presence of another double bond (Figure S28). The first is quaternary, and the second is attached to a proton resonating at 6.56 ppm, as shown in the HSQC spectra (Figure S9). In the HMBC spectrum, these protons show correlation with the carbonyl group at 201.2 ppm, the characteristic C-9, resonating at 131.5 ppm, the tertiary carbon at position 5 (48.3 ppm), and the methylene carbon at position 19 (25.4 ppm), suggesting that this proton is in position C-1 and the A ring has constructed (Figure S30). Using these new data in comparison with the spectral data of IMNA, the complete identification of compound **2** was achieved.

Similarly, the structural elucidation of compound **3**, based on its LC-ESI-HRMS data, revealed a molecular formula of C_30_H_46_O_5_, indicating the presence of two additional oxygen atoms compared to IMNA. The ^1^H NMR spectrum analysis revealed two peaks at 2.41 and 1.54 ppm, assigned to H-1, while a cross-peak between H-1 and C-2, resonating at 88.4 ppm, indicated a bond between C-2 and at least one oxygen atom (Figures S33 and S37). Notably, the ^13^C spectrum showed a prominent peak at 179.4 ppm, with the absence of the characteristic C-3 carbonyl peak at 218.3 ppm found in IMNA, suggesting the presence of a carboxyl group (Figure S35). This observation was further supported by the HMBC spectra, which demonstrated a correlation between this carbon and the protons at position C-1 (Figure S37). These protons are non-equivalent and resonate at different ppm values, 2.41 and 1.71 ppm, respectively. In the COSY spectrum, they exhibit interactions with each other, and in the HMBC spectrum, they show interactions with the quaternary carbon at position 2, which resonates at 88.4 ppm (Figures S34 and S37). The stereochemistry of these compounds aligns with the proposed mechanism and existing literature [35].

**Figure 3:**
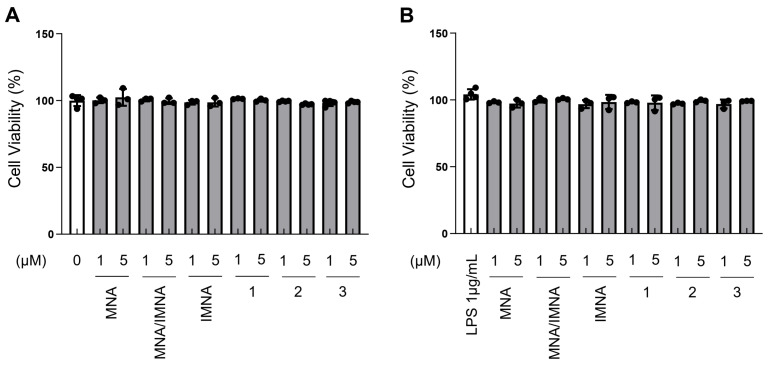
(**A**) Relative (%) survival (MTT assay) of RAW264.7 macrophages exposed to the indicated concentrations of the compounds for 24 h. (**B**) Cell viability of the compounds on LPS (1 µg/mL)-stimulated RAW264.7 macrophages. Control sample values were set to 100%. Bars, ±SD.

Building on the comprehensive structural characterization of IMNA, MNA, and their derivatives, the focus then shifted to the evaluation of their biological activities. Considering the established role of inflammation in various chronic diseases and existing studies highlighting CMG’s ability to reduce the levels of inflammatory markers, the potential of the MNA/IMNA mixture, along with pure MNA, IMNA, **1**, **2**, and **3**, was evaluated [36,37]. Before assessing the anti-inflammatory properties of the compounds, their impact on the viability of RAW264.7 cells was examined using the MTT assay. To this end, cells were incubated for 24 h with two different concentrations (1 and 5 μM) of compounds, and no cytotoxicity was observed (Figure 3A). Additionally, the cytotoxic effects of the compounds on lipopolysaccharide (LPS)-stimulated RAW264.7 cells were investigated. LPS, a component of the cell wall found in Gram-negative bacteria, plays a critical role in triggering the inflammatory response and contributing to the development of numerous inflammatory diseases [38]. The MTT assay showed that there were no significant cytotoxic effects under the treatment condition used in the study (Figure 3B).

**Figure 4:**
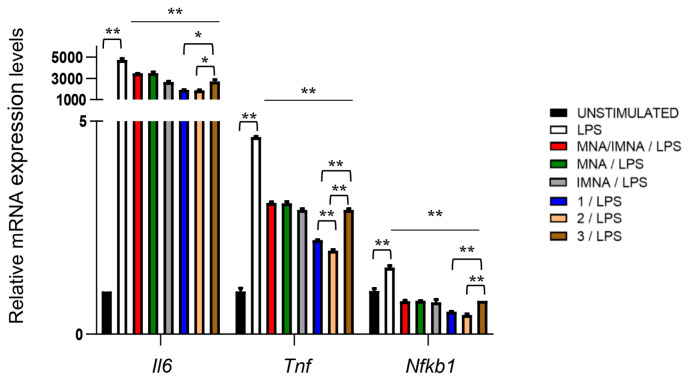
Relative mRNA expression levels of *Il6*, *TnF*, and *Nfkb1* in RAW 264.7 cells after LPS stimulation (1 μg/mL) and treatment using the compounds at 5 μM. Control values (unstimulated cells) were set to 1. Bars, ±SD; * *p* < 0.05; ** *p* < 0.01.

To assess the anti-inflammatory effect, various inflammation markers were analyzed using LPS-stimulated RAW 264.7 macrophage cells. Initially, the mRNA levels of *Il6*—key pro-inflammatory cytokine, as well as of *Tnf* which play a critical role in the onset and progression of inflammatory diseases—were measured. Additionally, the impact of the compounds on *Nfkb1* activation was investigated [39]. Co-treatment of macrophages stimulated by LPS (1 μg/mL) with the MNA/IMNA mixture, pure MNA, and IMNA and compounds **1**, **2,** and **3** (5 μM) resulted in milder induction of *Tnf*, *Il6*, and *Nfkb1* mRNA levels, which were significantly upregulated by LPS. Notably, MNA, IMNA, and their mixture exhibited similar effects against the tested inflammation markers. While all tested compounds demonstrated anti-inflammatory activity, compounds **1** and **2** showed the most promising effects, whereas compound **3** exhibited activity comparable to that of MNA and IMNA. These findings suggest that the *E*/*Z* configuration of the external double bond influences compound activity, with the E isomer showing increased activity. This insight guides the design of new analogs targeting the 24E isomer. Additionally, the α-hydroxy substitution on the α-carbonyl group of the A ring appears to enhance the activity of the tested compounds (Figure 4).

## 4. Discussion

This study addressed the limitations of existing methods for isolating MNA and IMNA from CMG by developing an efficient, high-yield, and straightforward approach. Conventional purification techniques often require multiple chromatographic steps and yield limited quantities [40,41]. In contrast, our optimized method enables the isolation of pure MNA and a high-yield mixture of MNA and IMNA in a single chromatographic step. Adjustments to the flow rate and gradient elution led to effective separation, making the process scalable and applicable to other triterpenic acids, such as moronic and oleanonic acids, in CMG.

A significant challenge of the current study was the selective production of IMNA in sufficient quantities, as conventional methods often fail to provide adequate purity. In order to address this, a reaction based on controlled migration of the internal double bond in MNA while minimizing undesired side reactions, such as further internal migration or *E*/*Z* isomerization of the external double bond, was developed. Systematic optimization of the reaction conditions—including acid strength, reagent equivalents, and reaction time—enabled the successful conversion of MNA to IMNA. This strategy also facilitated the isolation of a previously undescribed isomer (compound **1**), expanding the chemical variety of triterpenic acids derived from *Pistacia lentiscus*. Additionally, two novel compounds, with A-ring contraction, were obtained through the base-catalyzed transformation of IMNA, providing a novel scaffold for the development of new triterpene analogs.

Furthermore, the pharmacological evaluation highlights the significance of this work. The MNA/IMNA mixture, as well as pure MNA, IMNA, and compound **1**, demonstrated strong anti-inflammatory activity by significantly reducing *Tnf*, *Il6*, and *Nfkb1* mRNA levels in RAW 264.7 macrophage cells. IMNA and its synthetic *E* isomer exhibited superior activity, with compound **1** showing the highest potency. Additionally, the newly synthesized IMNA analogs (compounds **2** and **3**) displayed promising activity. Specifically, compound **3** demonstrated efficacy comparable to that of natural compounds, while compound **2** emerged as the most potent. These results underscore the potential for structural optimization to enhance therapeutic effects.

## 5. Conclusions

This streamlined approach ensures the recovery of pure MNA and the mixture of MNA/IMNA, facilitating subsequent in-depth exploration of their pharmacological properties. Treatment of the isomer mixture with BBr_3_ yields either the natural isomer IMNA or the *E* isomer **1**, in high yield, enabling a more focused analysis of their activities. Further investigation into the isomerization of the mixture under basic conditions led to the synthesis of two new compounds, featuring modifications to the A-ring of the triterpene core. Preliminary findings reveal promising anti-inflammatory properties for both the isomer mixture and the *Z*/*E* forms of IMNA, with the *E* isomer showing superior activity. This finding suggests that the configuration of the external double bond significantly influences the compound’s bioactivity, guiding the design of new analogs based on the 24*E* conformation. Moreover, the enhanced activity of compound **2** indicates that structural modifications to the A-ring, particularly the substitution at position 2, can improve efficacy against the tested inflammation markers.

To conclude, the isolation of these triterpenic acids and their analogs supports further evaluation against additional biological targets and facilitates the synthesis of new analogs intended to improve activity. Triterpenic structures emerge as a valuable scaffold for developing novel compounds, with the preliminary results highlighting the need for further investigation into their mechanisms of action. This continued research will clarify the structure–activity relationship, facilitating the design of more effective molecules with enhanced therapeutic potential.

## Data Availability

Data are contained within the article and Supplementary Materials.

## Supplementary Materials

The following supporting information can be downloaded at: https://www.mdpi.com/article/10.3390/biom14121618/s1, Scheme of the procedure of the pilot extraction, HPLC chromatograms, mass spectra of all compounds, as well as 1D and 2D NMR experiments are available online.
