# Supplementary material for: Anti-Inflammatory Activity of the Major Triterpenic Acids of Chios Mastic Gum and Their Semi-Synthetic Analogues
Source: Biomolecules. 2024 Dec 18;14(12):1618. doi: 10.3390/biom14121618 (PMC11727566; doi:10.3390/biom14121618)
Supplement: Supplementary file 1 [file biomolecules-14-01618-s001.zip › biomolecules-3365492-supplementary.pdf]

Supplementary Materials

Contents

Figure S1: Procedure of the pilot extraction of the **NF** and **AF** of triterpenes of CMG. 1

Figure S2: Chromatogram of the acidic fraction of triterpenes using RP-HPLC with an ELSD detector.........1

Figure S3: Chromatogram of the isolation of MNA and the mixture MNA/IMNA using preparative HPLC-DAD. 1

Figure S4: Mass spectra of **MNA.** 2

Figure S5: ^{1}H (400 MHz, $CDCl_3$) spectrum of **MNA**. 3

Figure S6: COSY (400 MHz, $CDCl_3$) spectrum of **MNA**. 3

Figure S7: ^{13}C (100 MHz, $CDCl_3$) spectrum of **MNA**..........4

Figure S8: HSQC-DEPT (400 MHz, $CDCl_3$) spectrum of **MNA**. 5

Figure S9: HMBC (400 MHz, $CDCl_3$) spectrum of **MNA**. 6

Figure S10: NOESY (600 MHz, $CDCl_3$) spectrum of **MNA**. 6

Figure S11: Mass spectra of **IMNA**..........7

Figure S12: ^{1}H (600 MHz, $CDCl_3$) spectrum of **IMNA**..........8

Figure S13: COSY (600 MHz, $CDCl_3$) spectrum of **IMNA**..........8

Figure S14: ^{13}C (151 MHz, $CDCl_3$) spectrum of **IMNA**. 9

Figure S15: HSQC-DEPT (600 MHz, $CDCl_3$) spectrum of **IMNA**. 10

Figure S16: HMBC (600 MHz, $CDCl_3$) spectrum of **IMNA**. 11

Figure S17: NOESY (600 MHz, $CDCl_3$) spectrum of **IMNA**..........11

Figure S18: Mass spectra of compound **1**..........12

Figure S19: ^{1}H (600 MHz, $CDCl_3$) spectrum of **1**..........13

Figure S20: COSY (600 MHz, $CDCl_3$) spectrum of **1**. 13

Figure S21: ^{13}C (151 MHz, $CDCl_3$) spectrum of **1**. 14

Figure S22: HSQC-DEPT (600 MHz, $CDCl_3$) spectrum of **1**. 15

Figure S23: HMBC (600 MHz, $CDCl_3$) spectrum of **1**. 16

Figure S24: NOESY (600 MHz, $CDCl_3$) of **1**. 16

Figure S25: Mass spectra of **2**..........17

Figure S26:^{1}H (600 MHz, $CDCl_3$) spectrum of **2**..........18

Figure S27: COSY (600 MHz$CDCl_3$) spectrum of **2**. 18

Figure S28: ^{13}C (151 MHz, $CDCl_3$) spectrum of **2**. 19

Figure S29: HSQC-DEPT (600 MHz, $CDCl_3$) spectrum of **2**. 20

Figure S30: HMBC (600 MHz, $CDCl_3$) spectrum of **2**. 21

Figure S31: NOESY (600 MHz, $CDCl_3$) spectrum of **2**..........21

Figure S32: Mass spectra of **3**..........22

Figure S33:^{1}H (600 MHz, MeOD) spectrum of **3**..........23

Figure S34: COSY (600 MHz, MeOD) spectrum of **3**. 23
Figure S35: ^{13}C (151 MHz, MeOD) spectrum of **3**. 24
Figure S36: HSQC-DEPT (600 MHz, MeOD) spectrum of **3**. 25
Figure S37: HMBC (600 MHz, MeOD), spectrum of **3**. 26
Figure S38: NOESY (600 MHz, MeOD) spectrum of **3**. 26

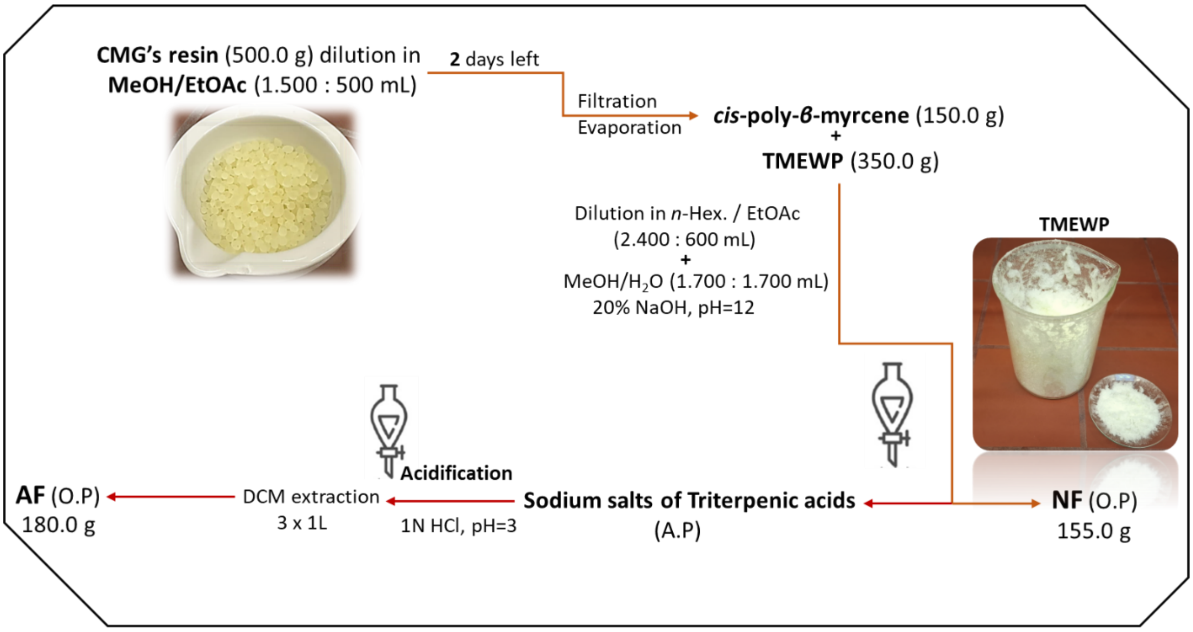

Figure S1: Procedure of the pilot extraction of the **NF** and **AF** of triterpenes of CMG.

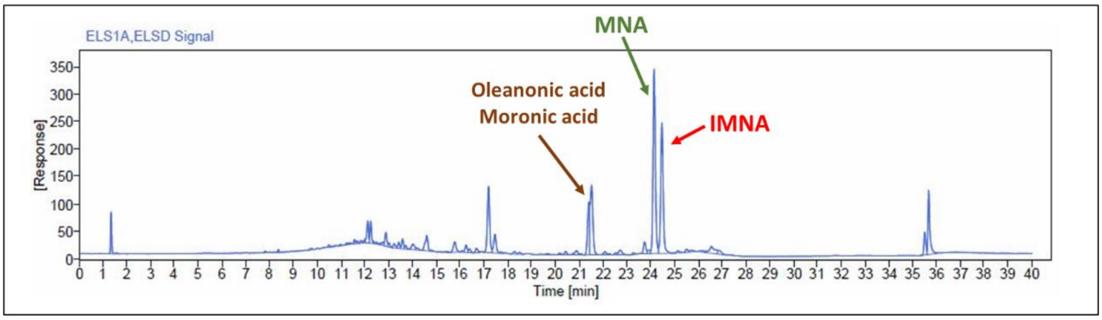

Figure S2: Chromatogram of the acidic fraction of triterpenes using RP-HPLC with an ELSD detector.

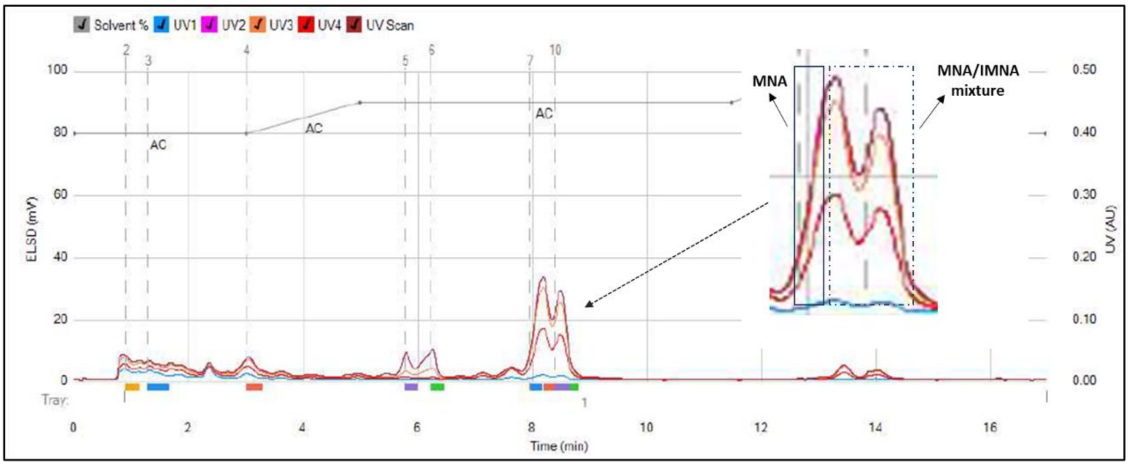

Figure S3: Chromatogram of the isolation of MNA and the mixture MNA/IMNA using preparative HPLC-DAD.

24*Z*-masticadienonic acid (MNA)

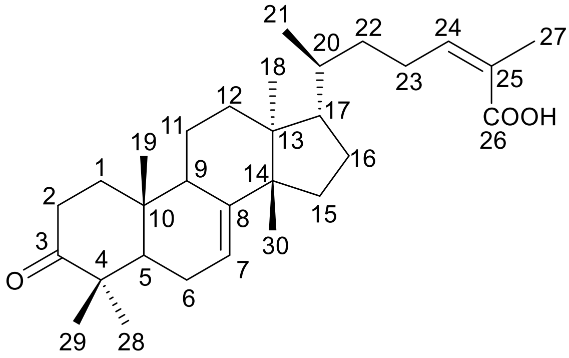

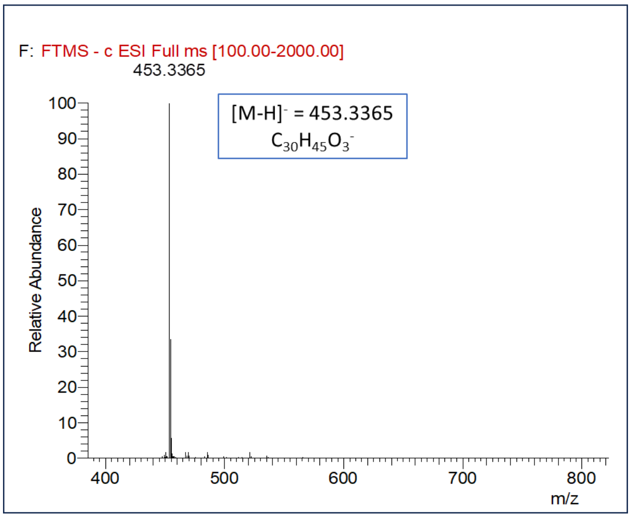

Figure S4: Mass spectra of **MNA.**

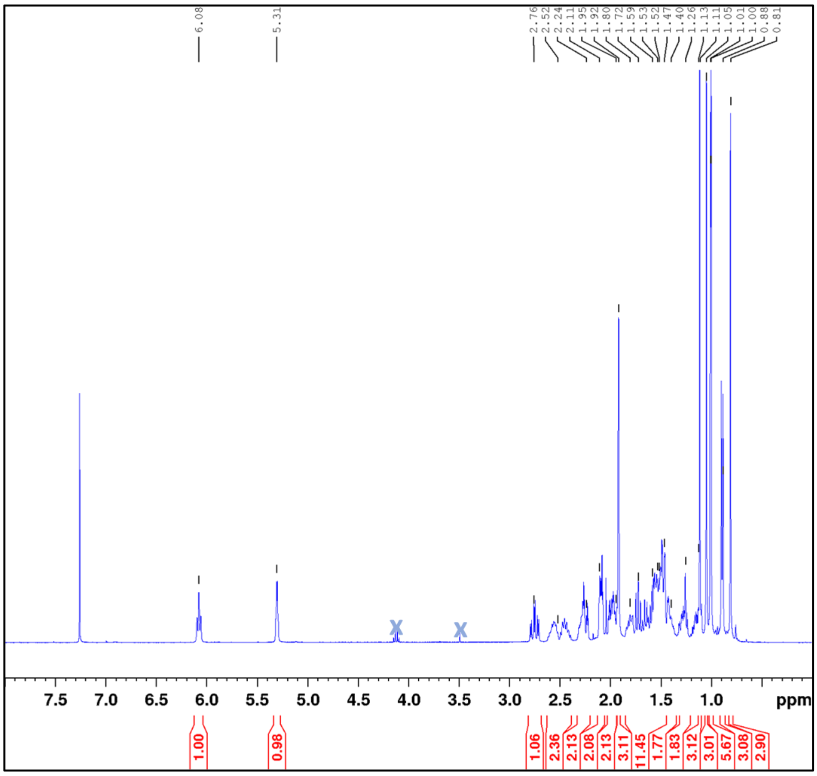

Figure S5: ^{1}H (400 MHz, $CDCl_3$) spectrum of **MNA**.

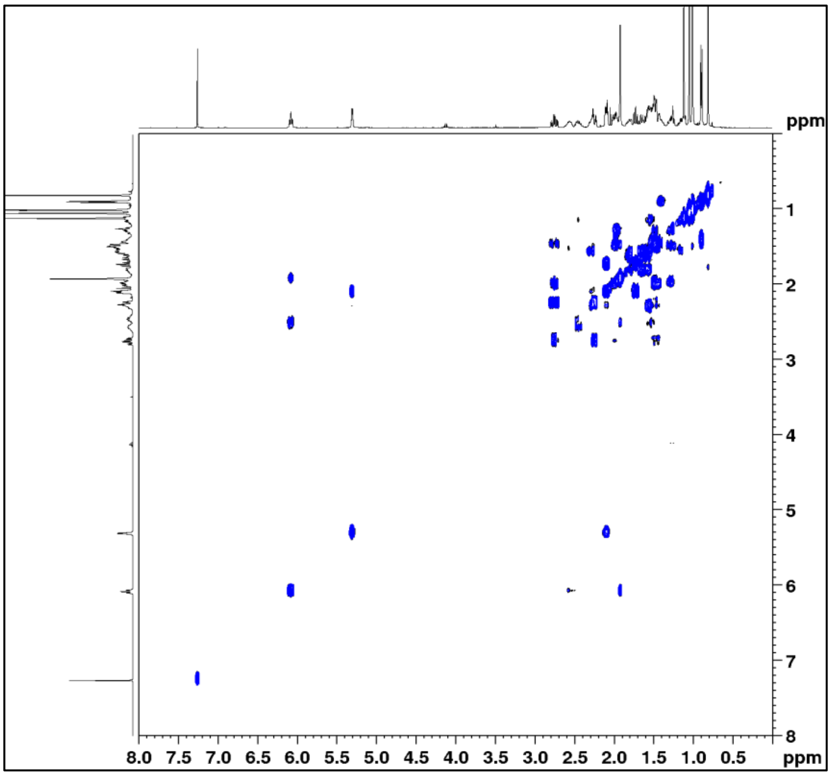

Figure S6: COSY (400 MHz, $CDCl_3$) spectrum of **MNA**.

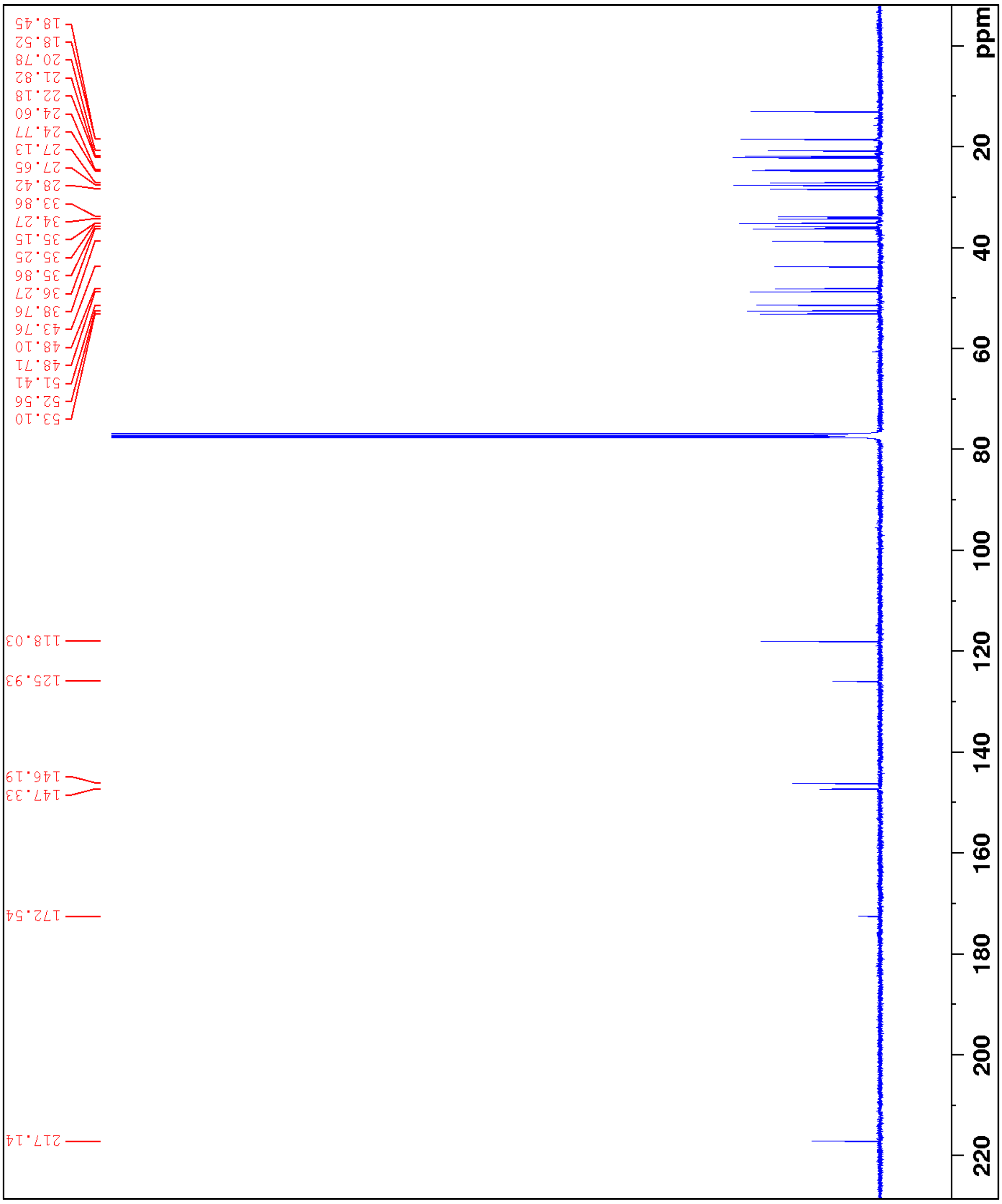

Figure S7: ^{13}C (100 MHz, $CDCl_3$) spectrum of **MNA**.

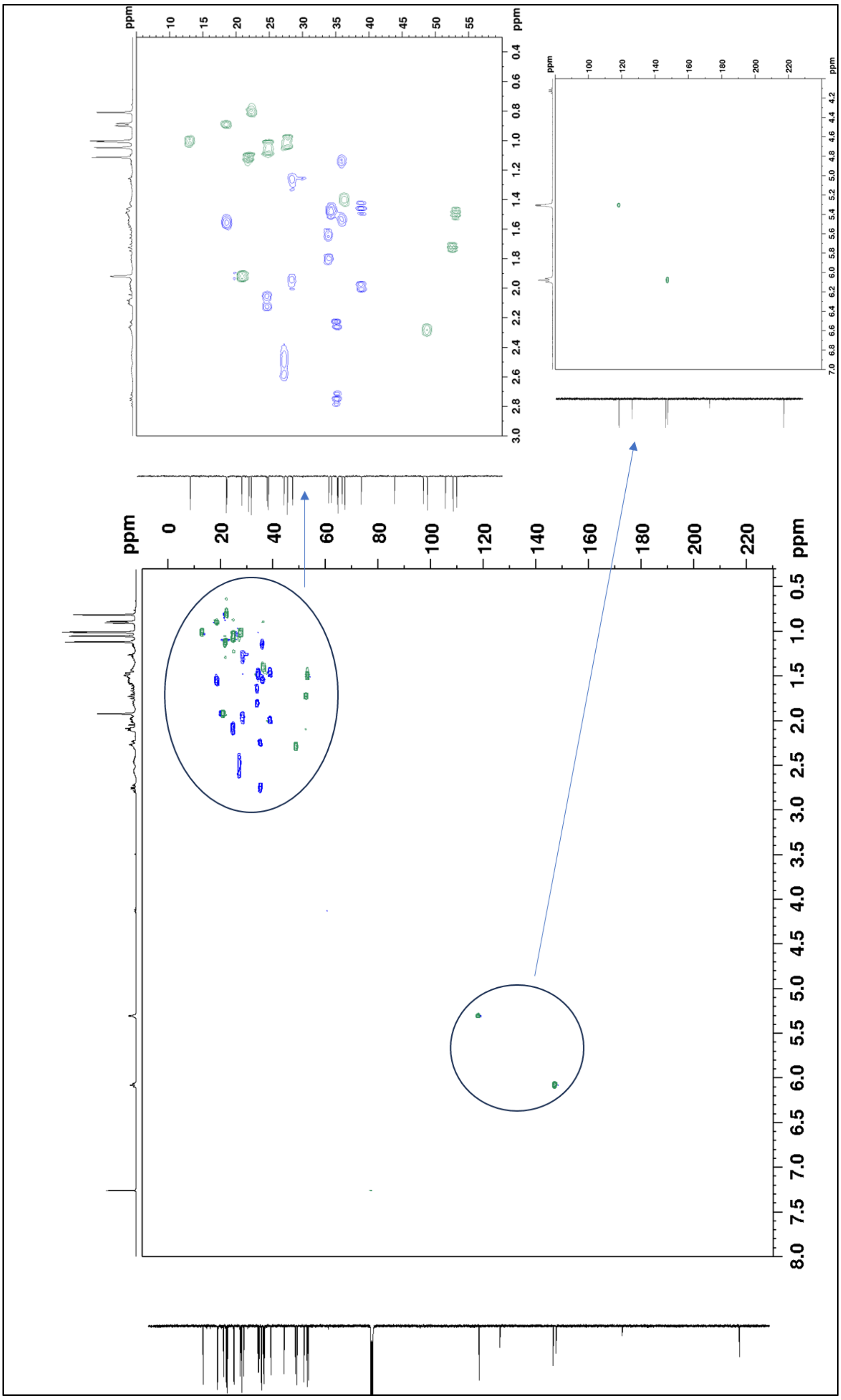

Figure S8: HSQC-DEPT (400 MHz, $CDCl_3$) spectrum of **MNA**.

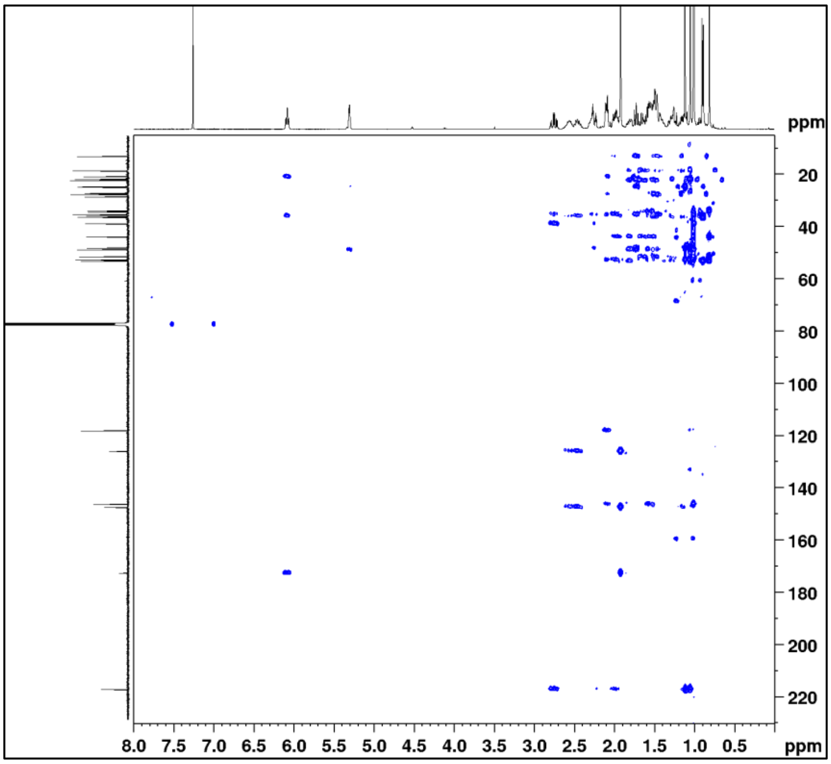

Figure S9: HMBC (400 MHz, $CDCl_3$) spectrum of **MNA**.

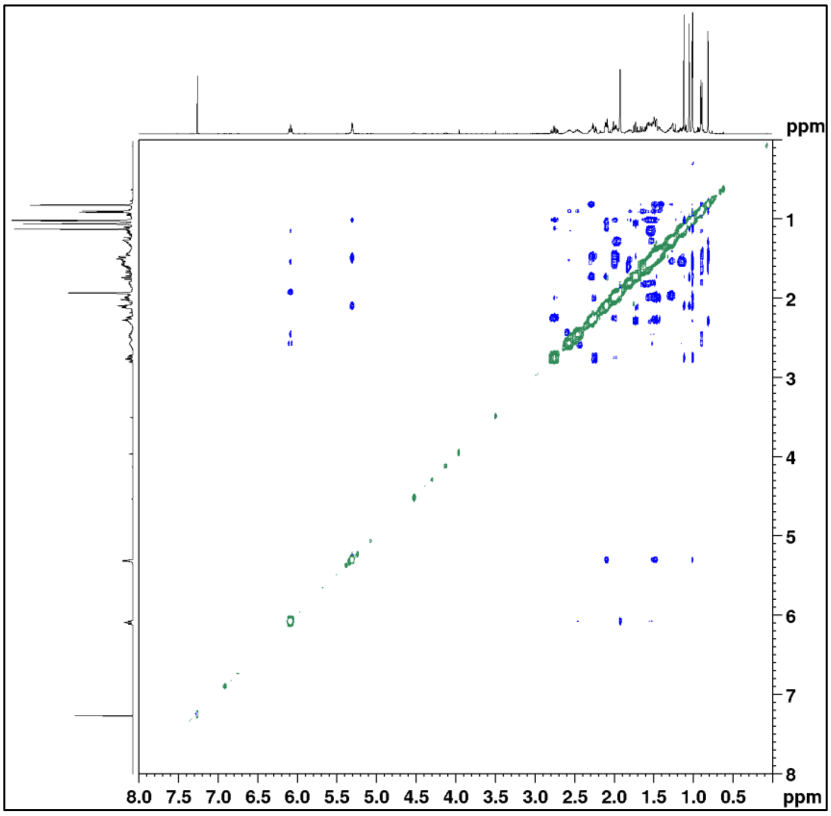

Figure S10: NOESY (600 MHz, $CDCl_3$) spectrum of **MNA**.

24*Z*-isomasticadienonic acid (IMNA)

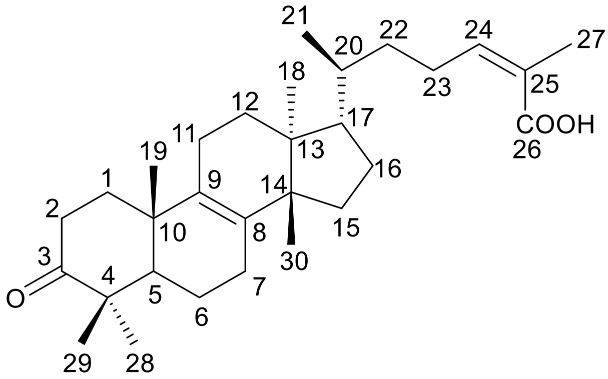

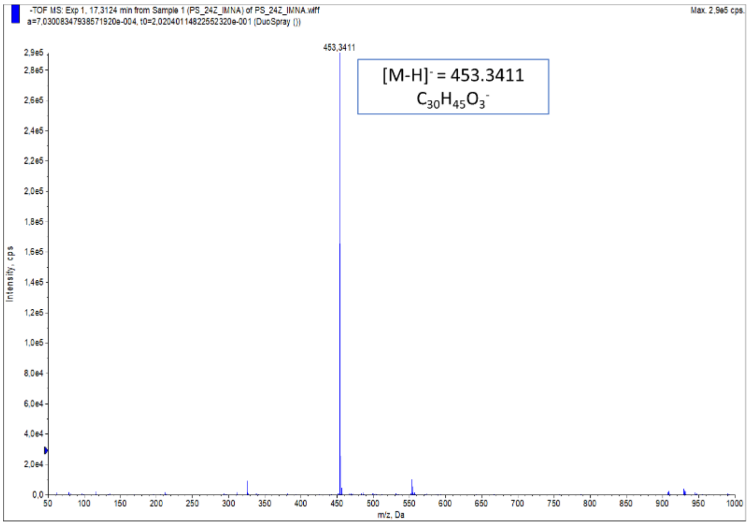

Figure S11: Mass spectra of **IMNA**.

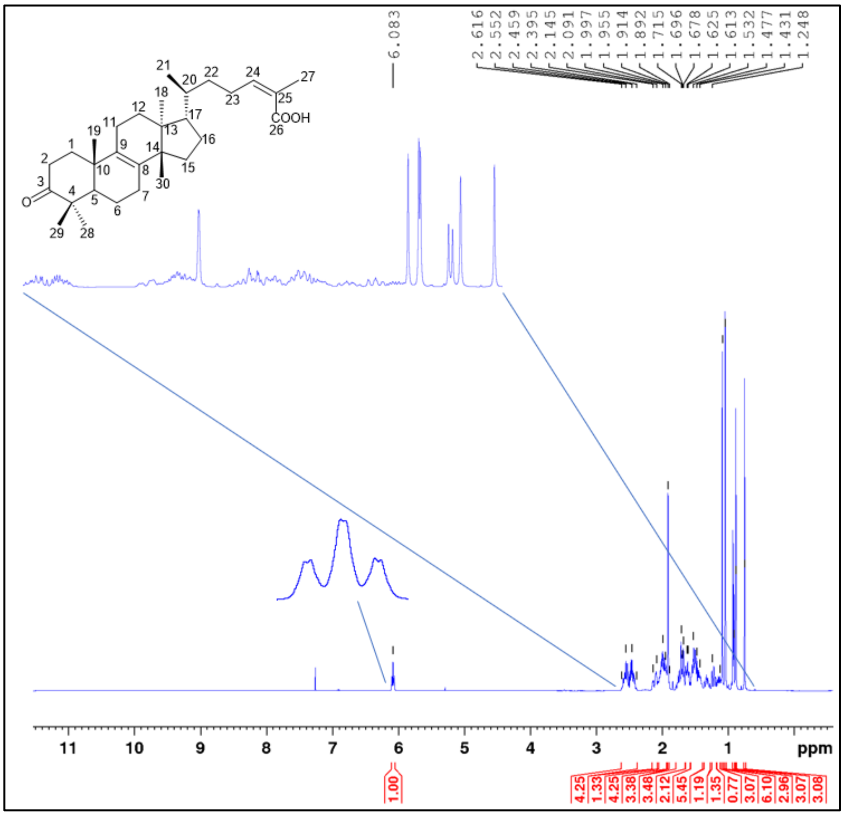

Figure S12: ^{1}H (600 MHz, $CDCl_3$) spectrum of **IMNA**.

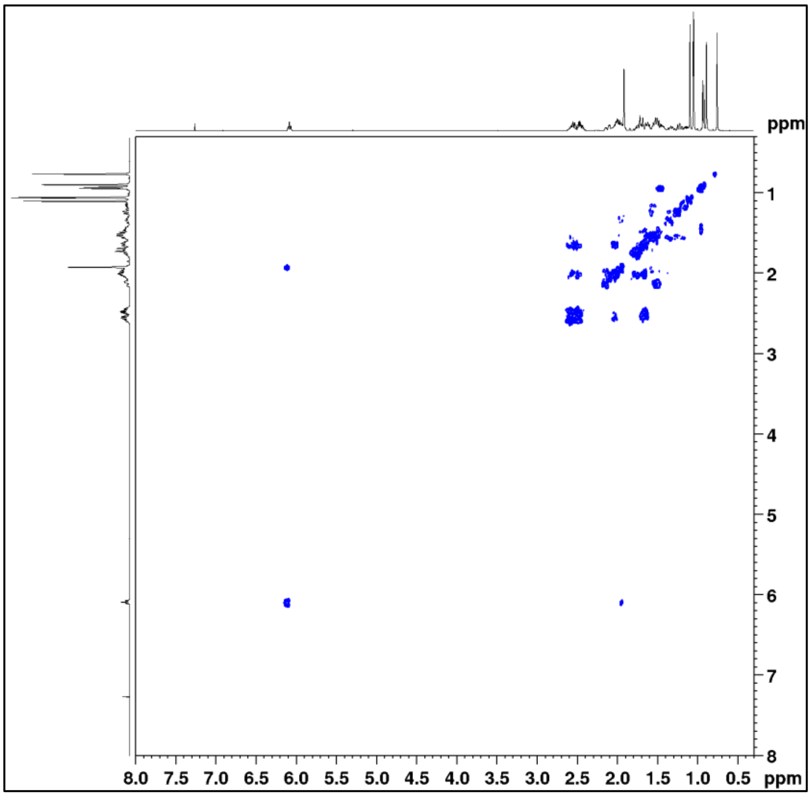

Figure S13: COSY (600 MHz, $CDCl_3$) spectrum of **IMNA**.

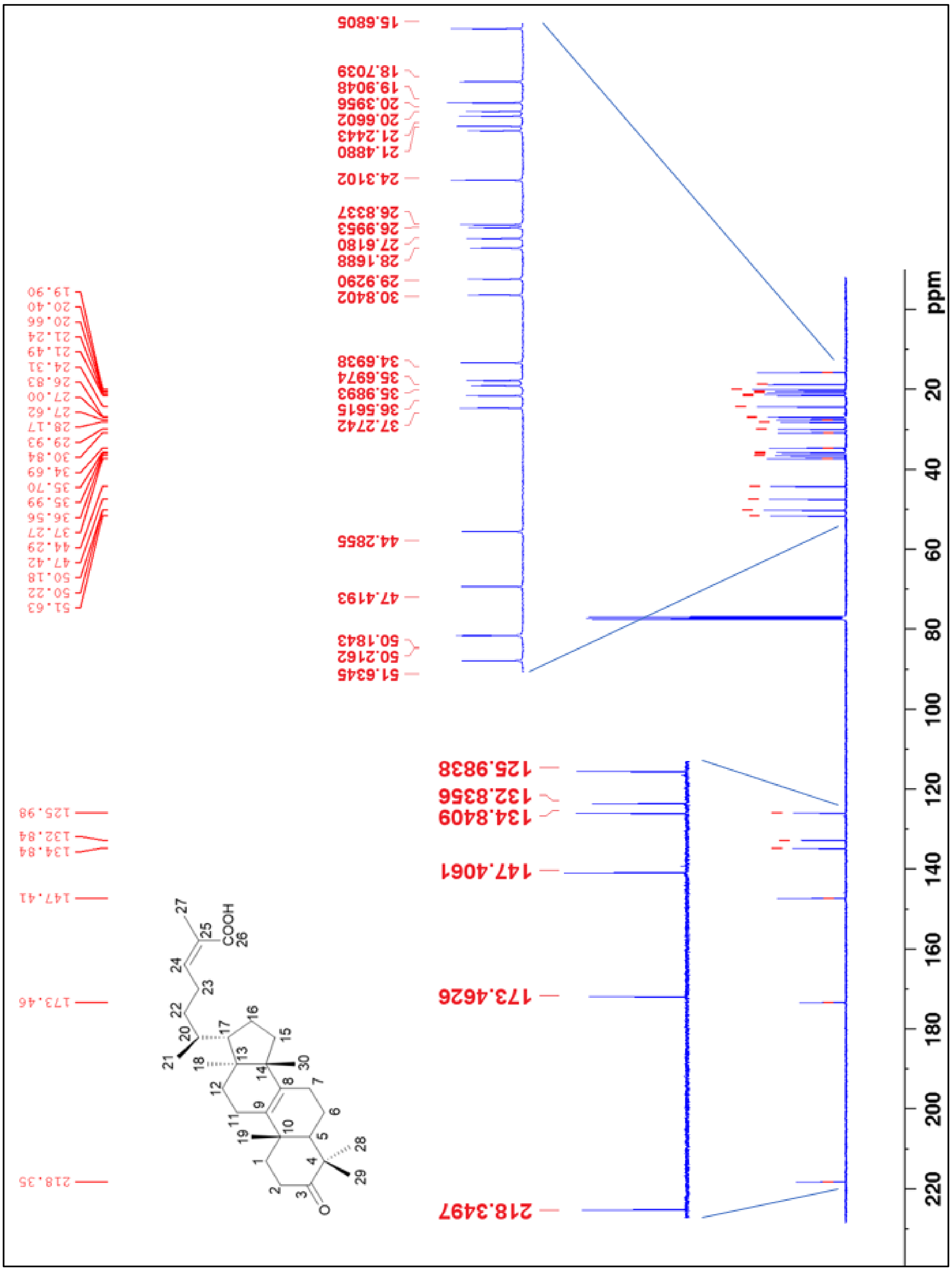

Figure S14: ^{13}C (151 MHz, $CDCl_3$) spectrum of **IMNA**.

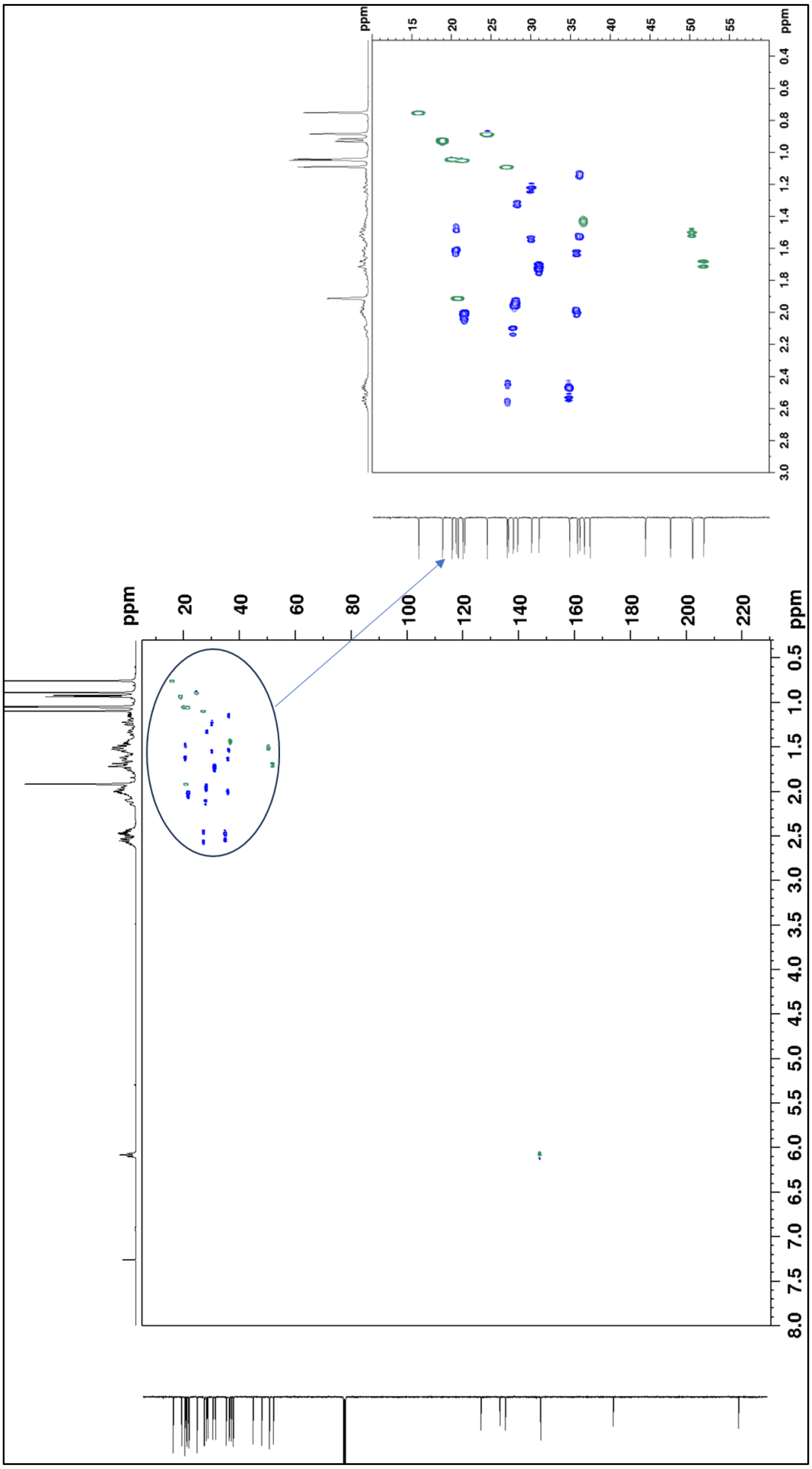

Figure S15: HSQC-DEPT (600 MHz, $CDCl_3$) spectrum of **IMNA**.

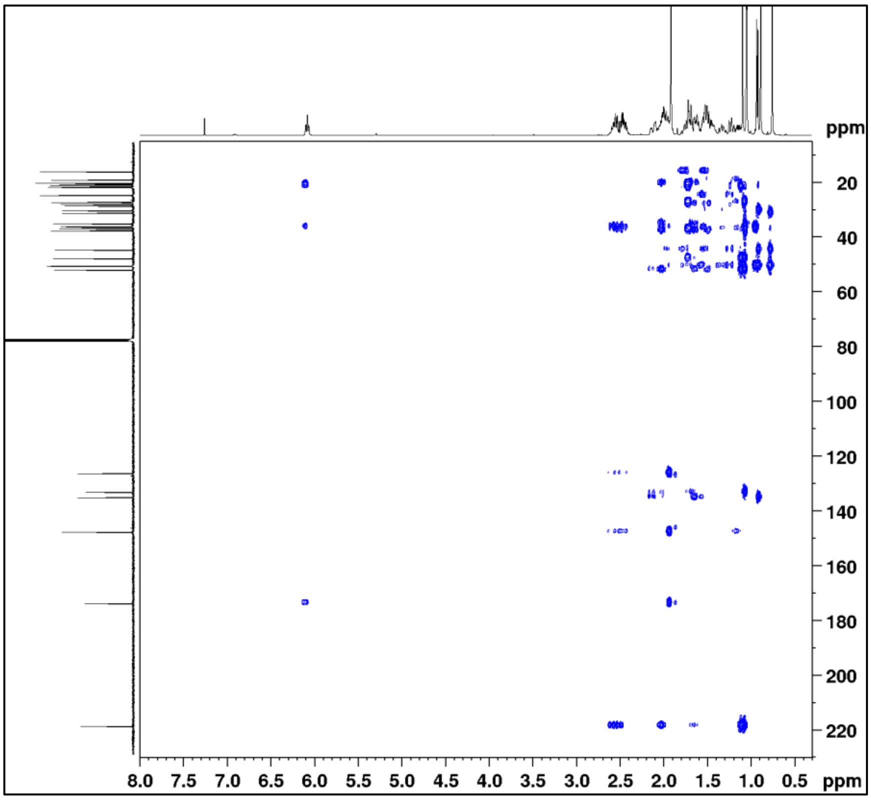

Figure S16: HMBC (600 MHz, $CDCl_3$) spectrum of **IMNA**.

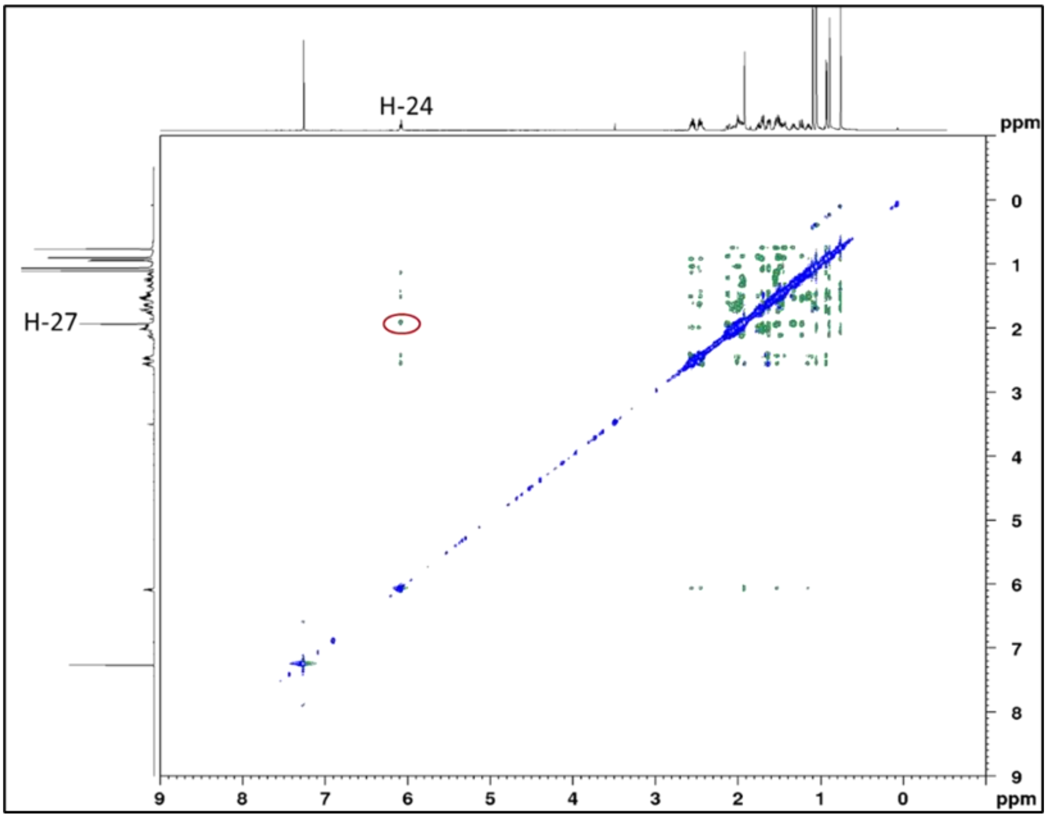

Figure S17: NOESY (600 MHz, $CDCl_3$) spectrum of **IMNA**.

24*E*-isomasticadienonic acid (1)

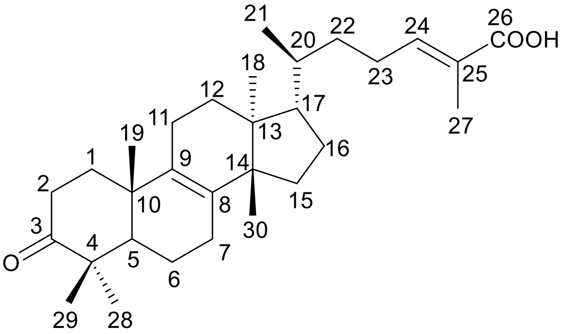

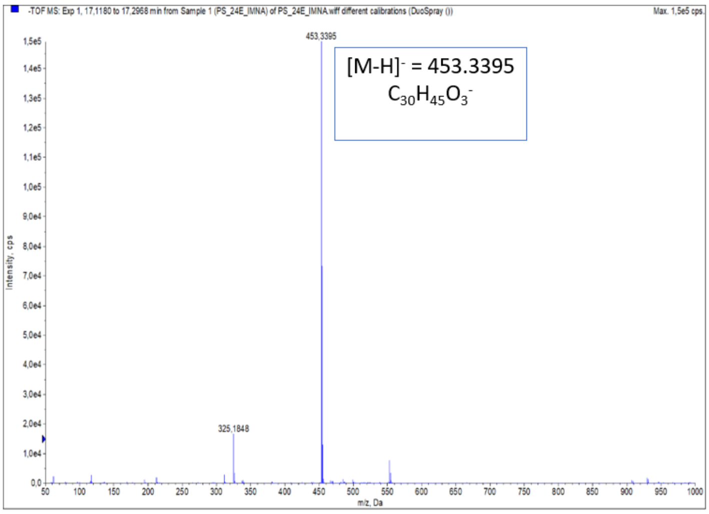

Figure S18: Mass spectra of compound **1**.

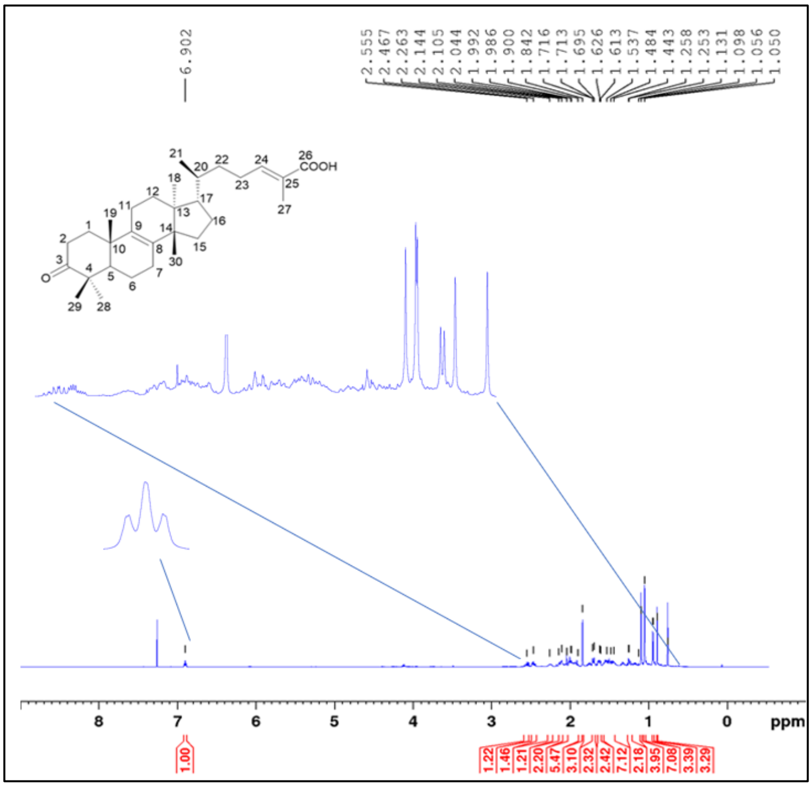

Figure S19: ^{1}H (600 MHz, $CDCl_3$) spectrum of **1**.

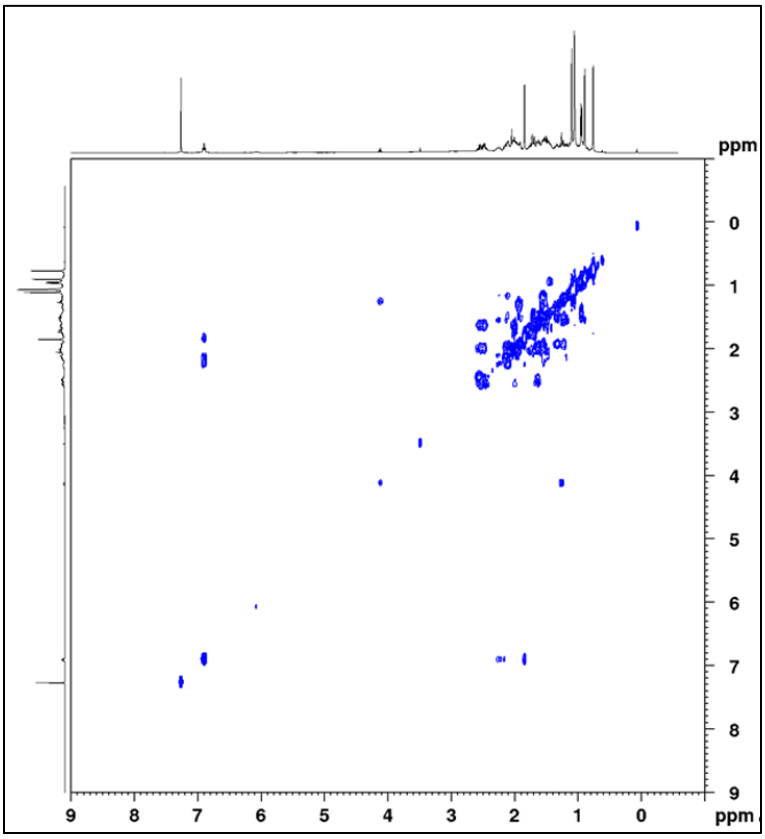

Figure S20: COSY (600 MHz, $CDCl_3$) spectrum of **1**.

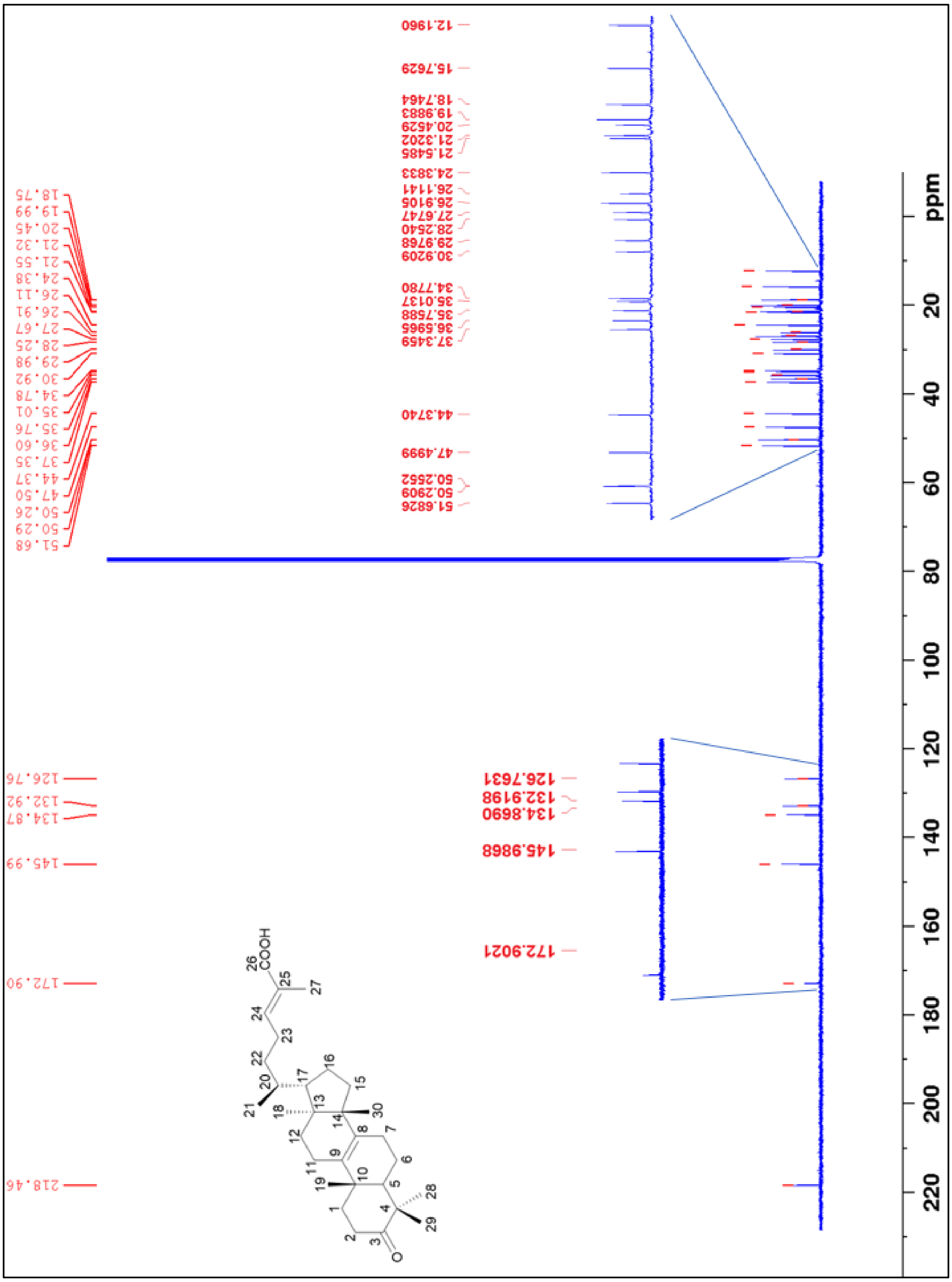

Figure S21: ^{13}C (151 MHz, $CDCl_3$) spectrum of **1**.

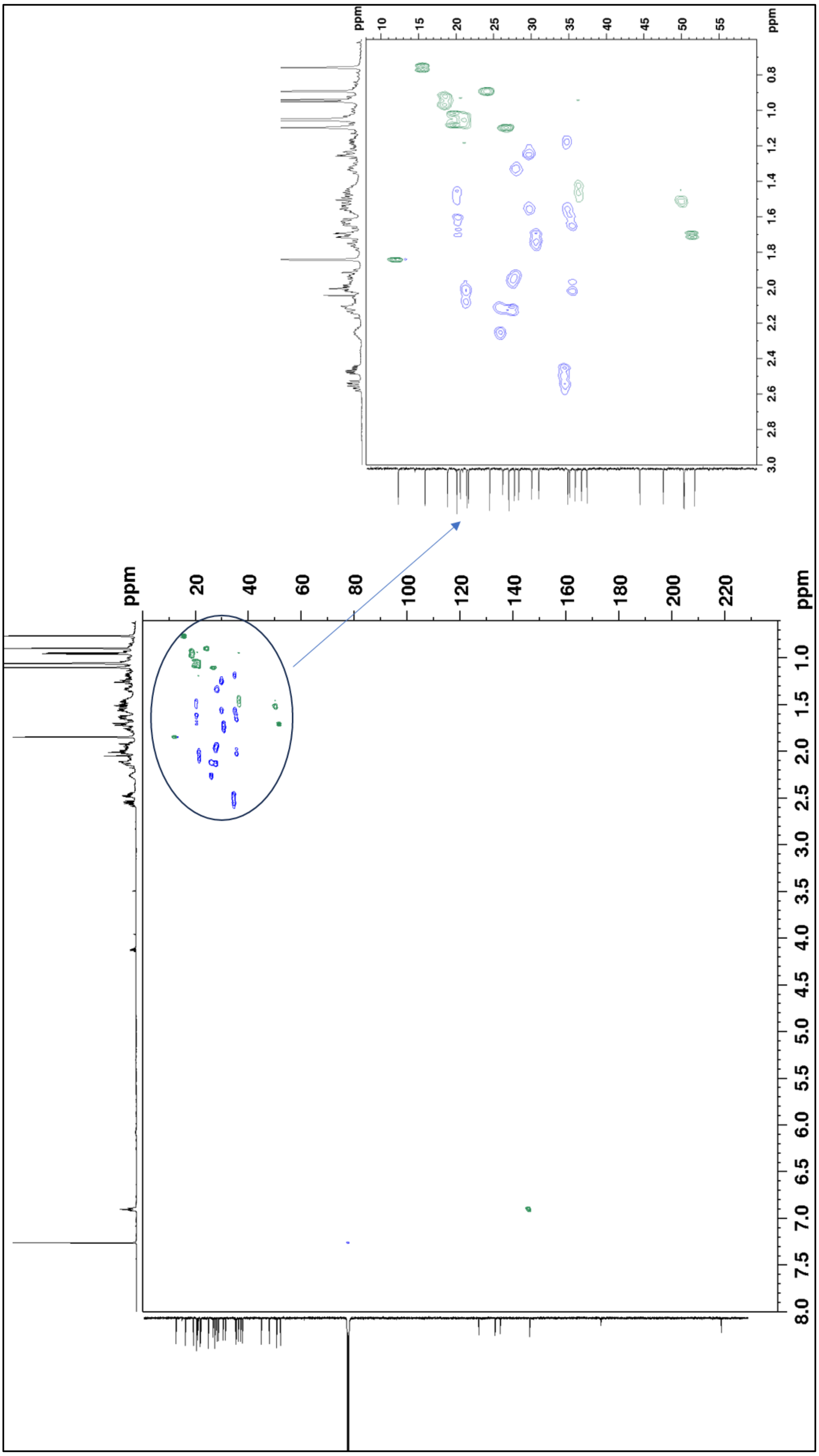

Figure S22: HSQC-DEPT (600 MHz, $CDCl_3$) spectrum of **1**.

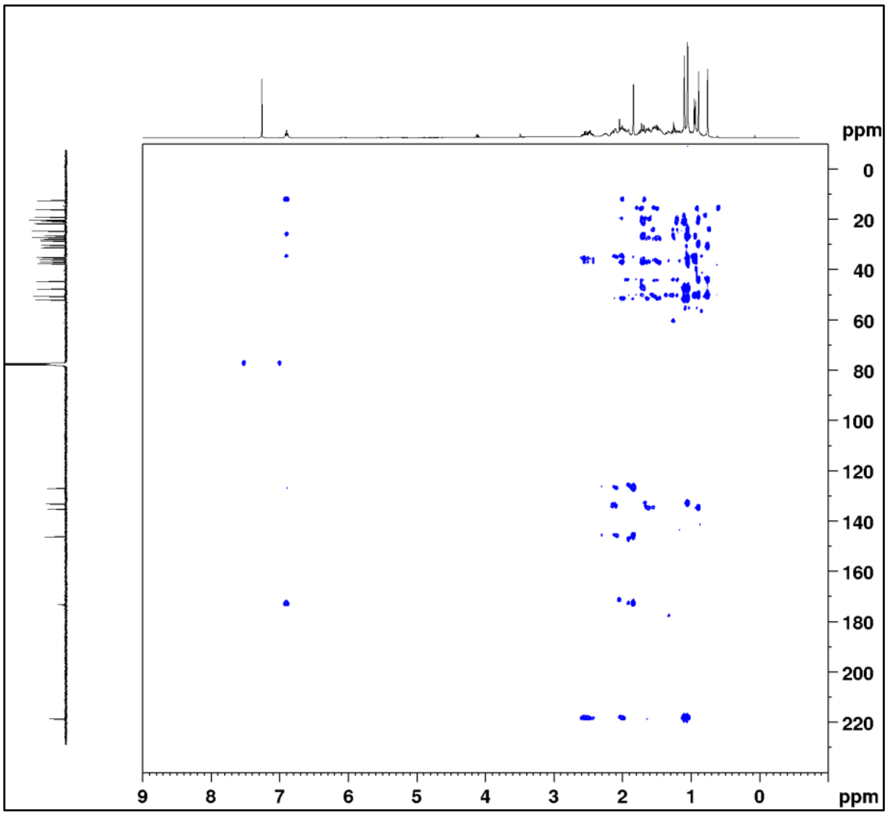

Figure S23: HMBC (600 MHz, $CDCl_3$) spectrum of **1**.

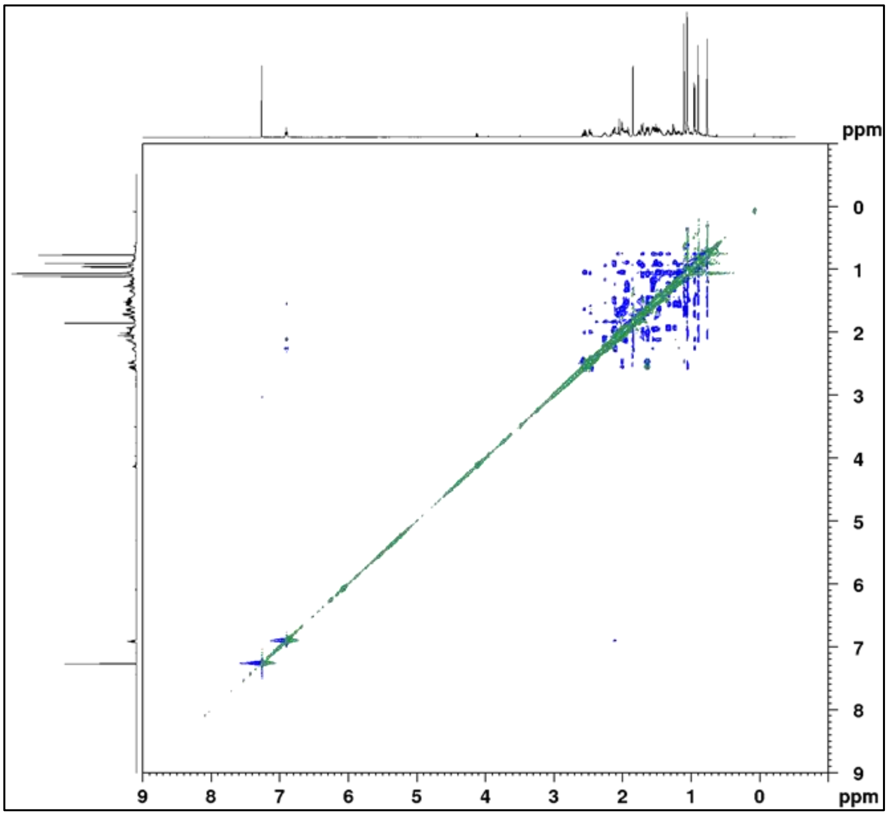

Figure S24: NOESY (600 MHz, $CDCl_3$) of **1**.

24Z-2-hydroxy-3-oxotirucalla-1,8,24-trien-26-oic acid (2)

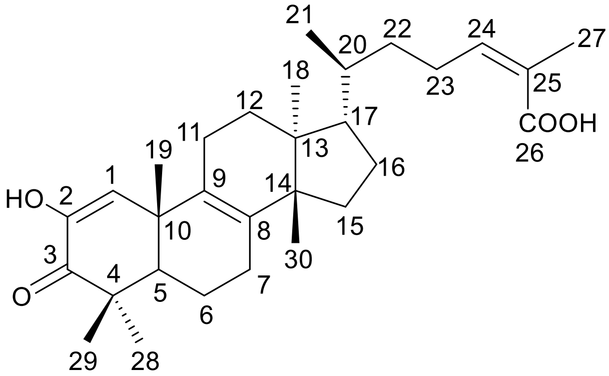

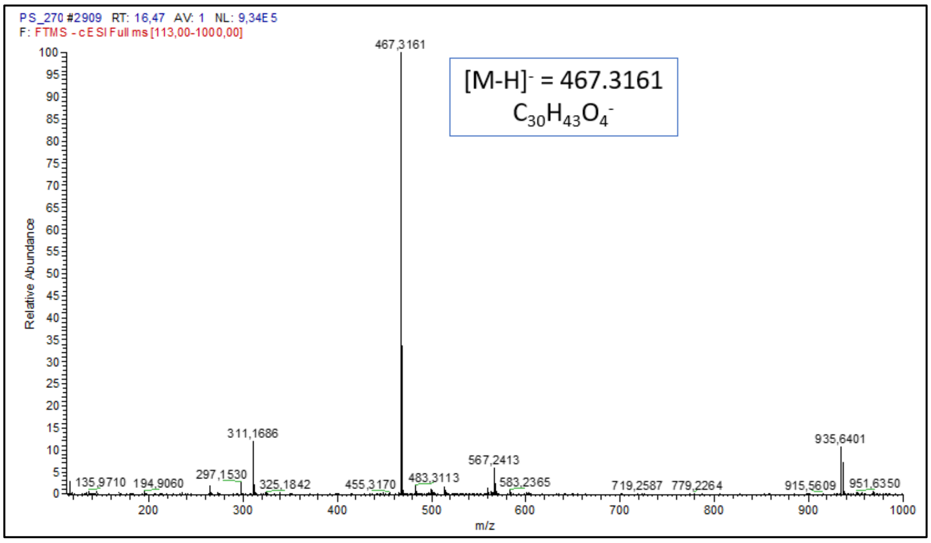

Figure S25: Mass spectra of **2**.

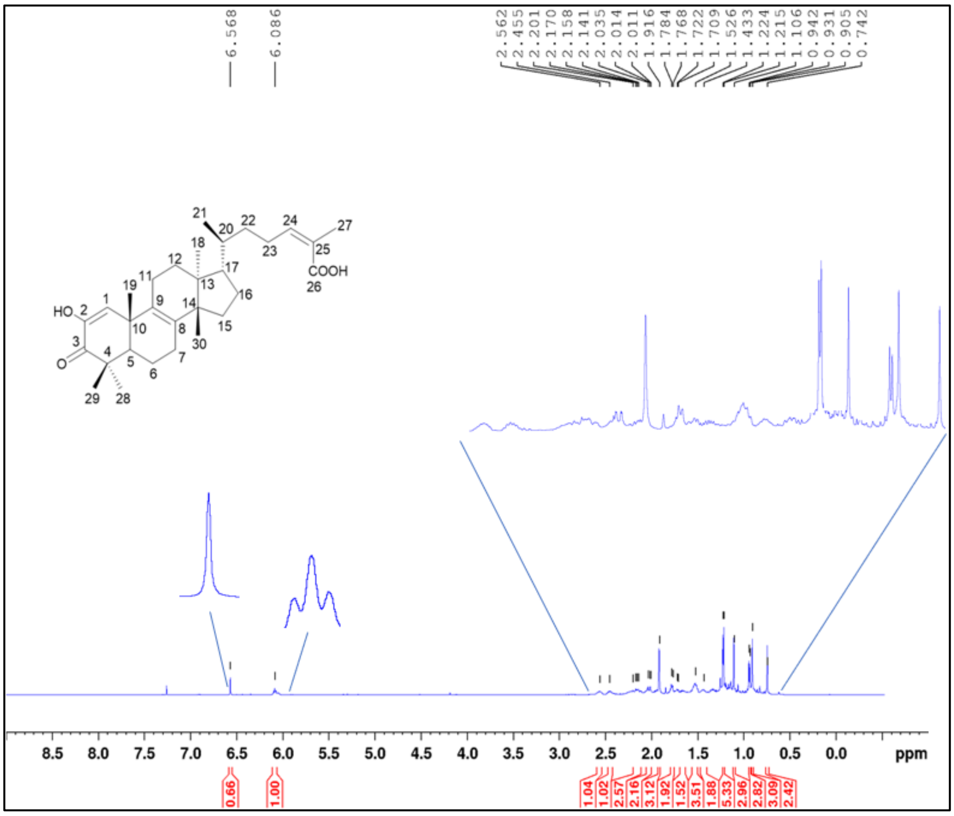

Figure S26:¹H (600 MHz, $CDCl_3$) spectrum of **2**.

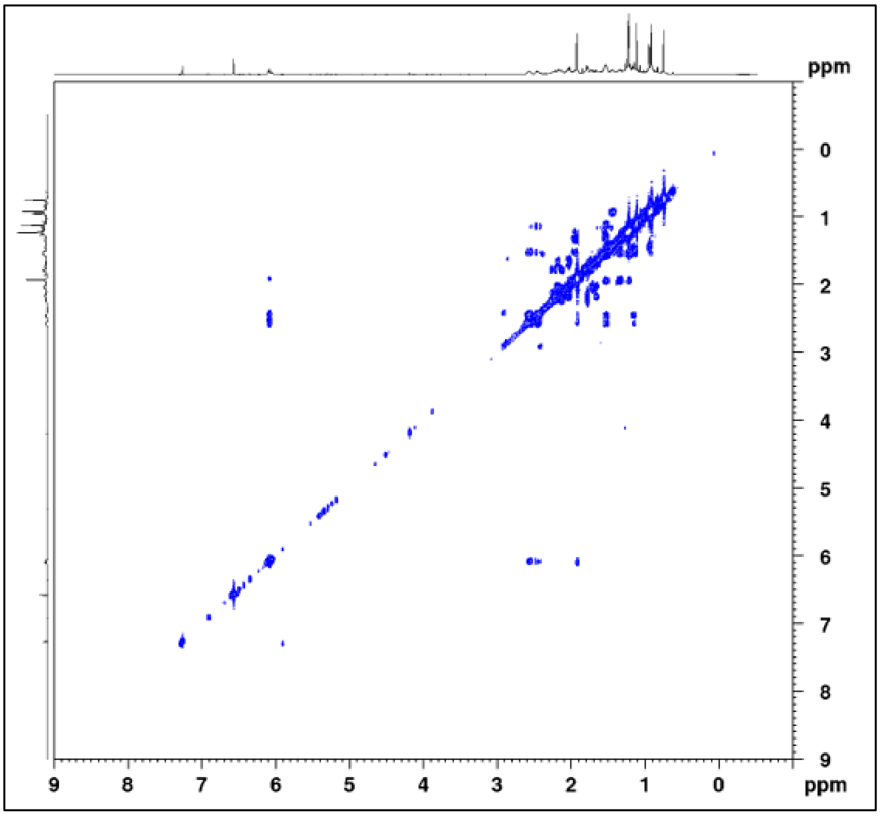

Figure S27: COSY (600 MHzCDCl₃) spectrum of **2**.

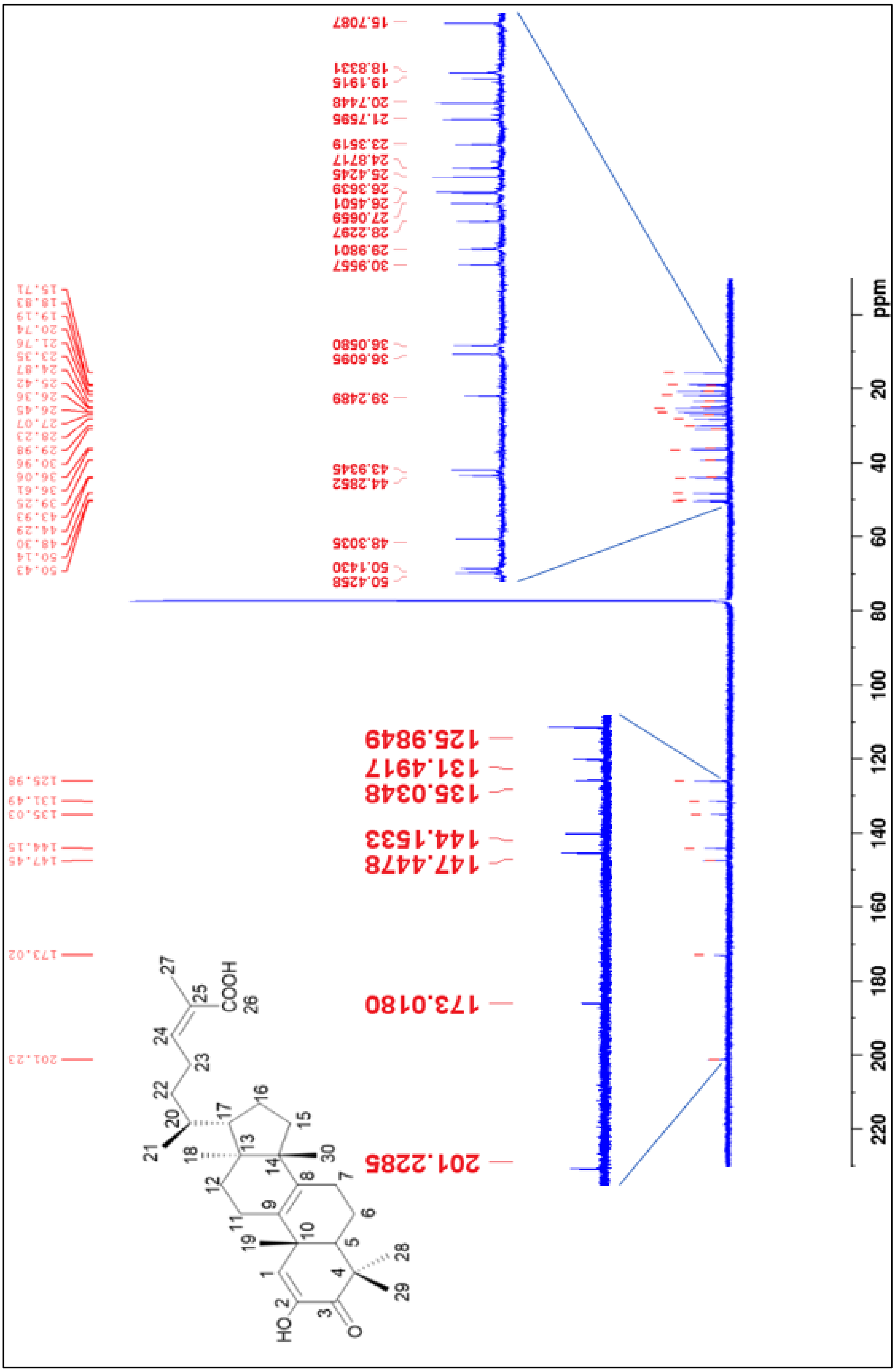

Figure S28: ^{13}C (151 MHz, $CDCl_3$) spectrum of **2**.

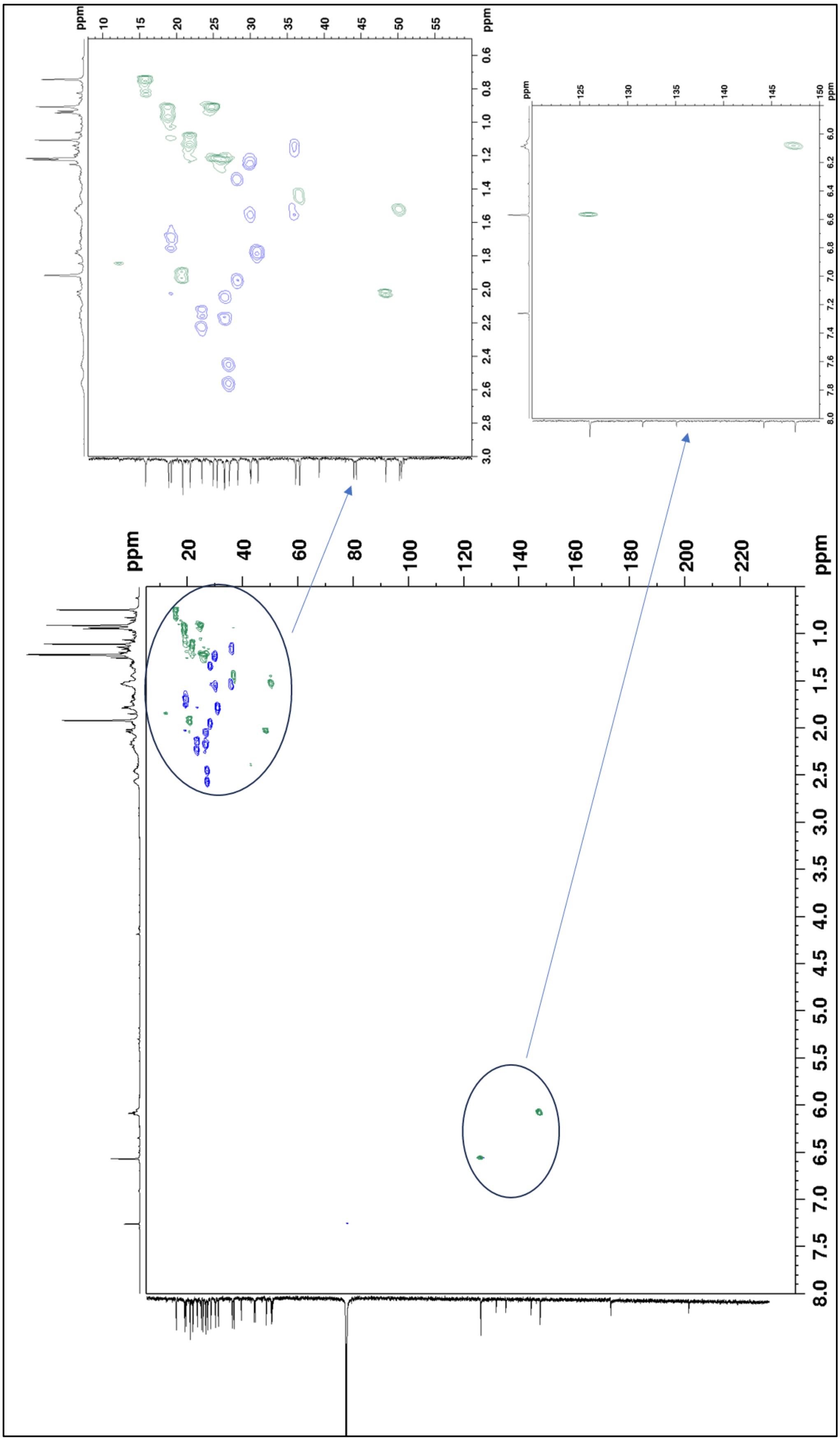

Figure S29: HSQC-DEPT (600 MHz, $CDCl_3$) spectrum of **2**.

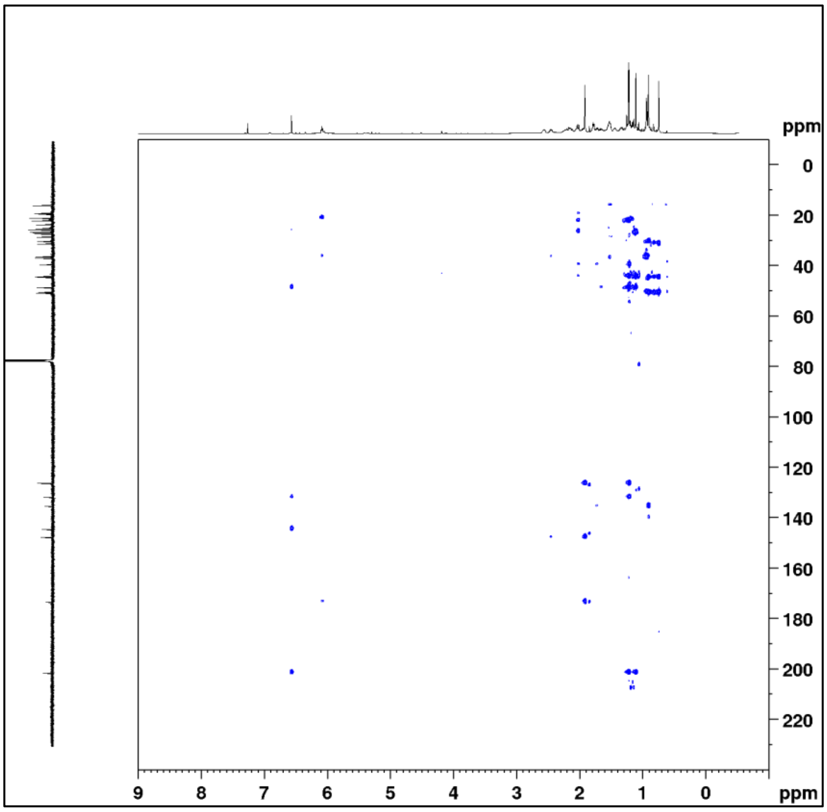

Figure S30: HMBC (600 MHz, $CDCl_3$) spectrum of **2**.

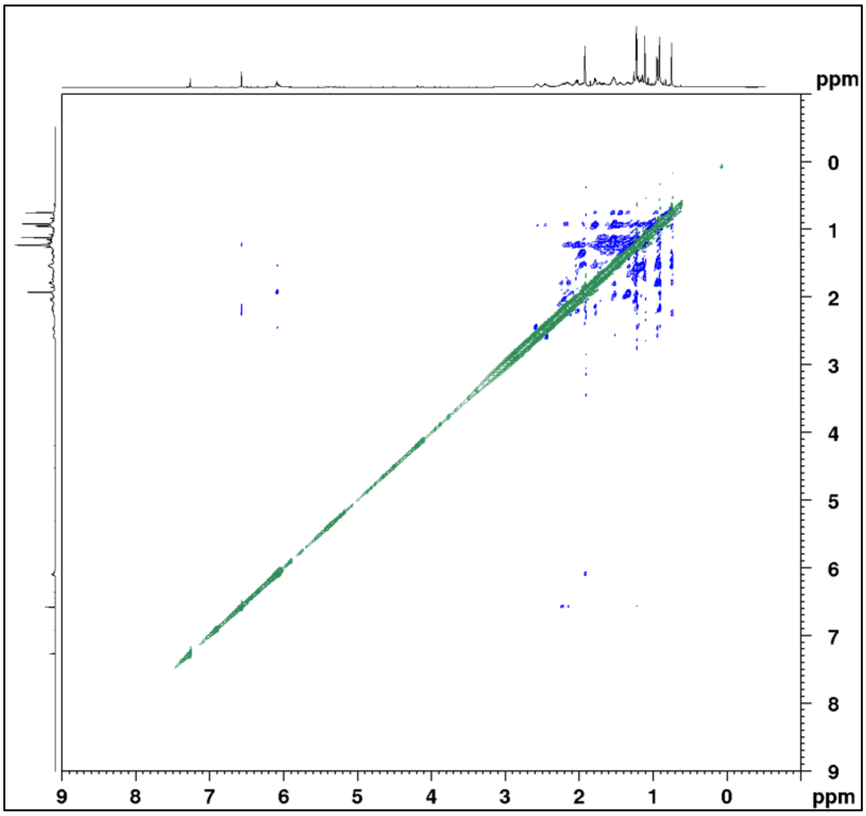

Figure S31: NOESY (600 MHz, $CDCl_3$) spectrum of **2**.

24*Z*-3b-hydroxy-1(2→3)-abeotirucalla-8,24-dien-2,26-dioic acid (3)

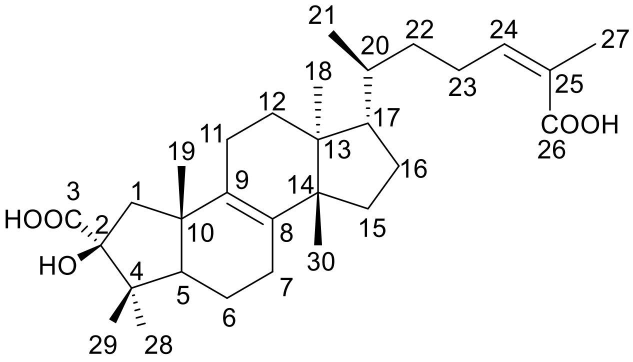

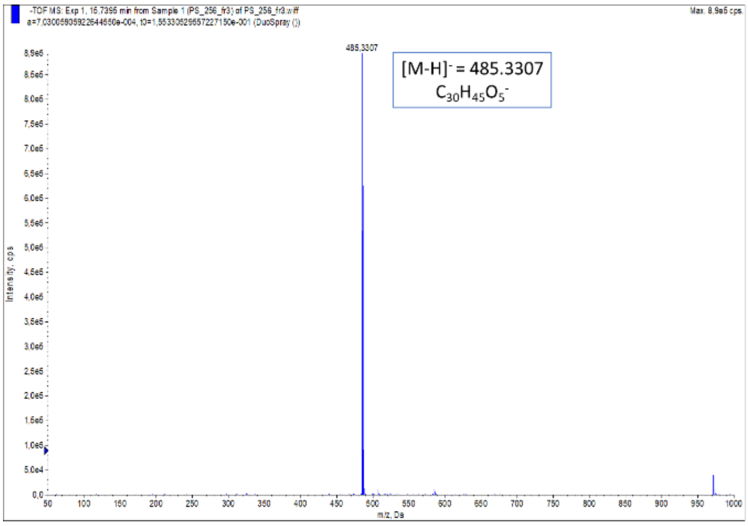

Figure S32: Mass spectra of **3**.

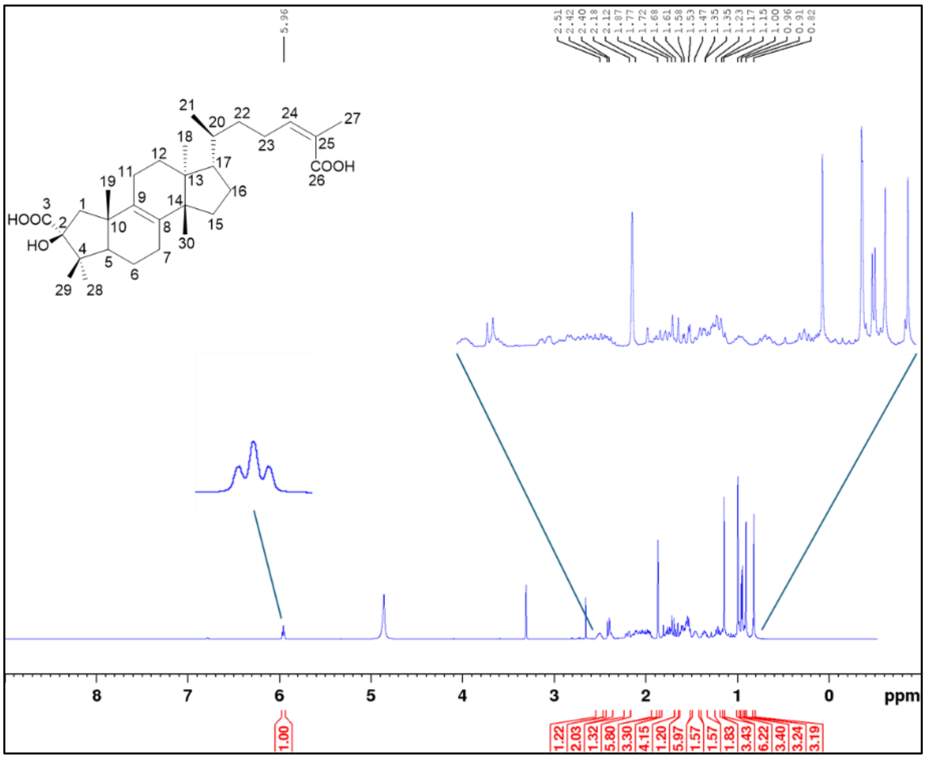

Figure S33:^{1}H (600 MHz, MeOD) spectrum of **3**.

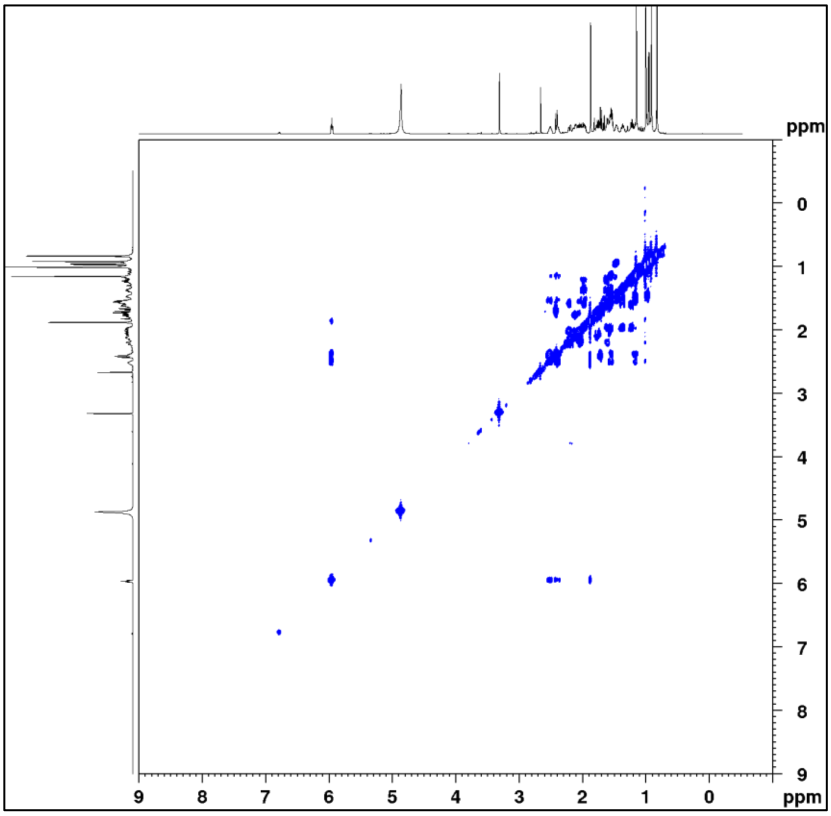

Figure S34: COSY (600 MHz, MeOD) spectrum of **3**.

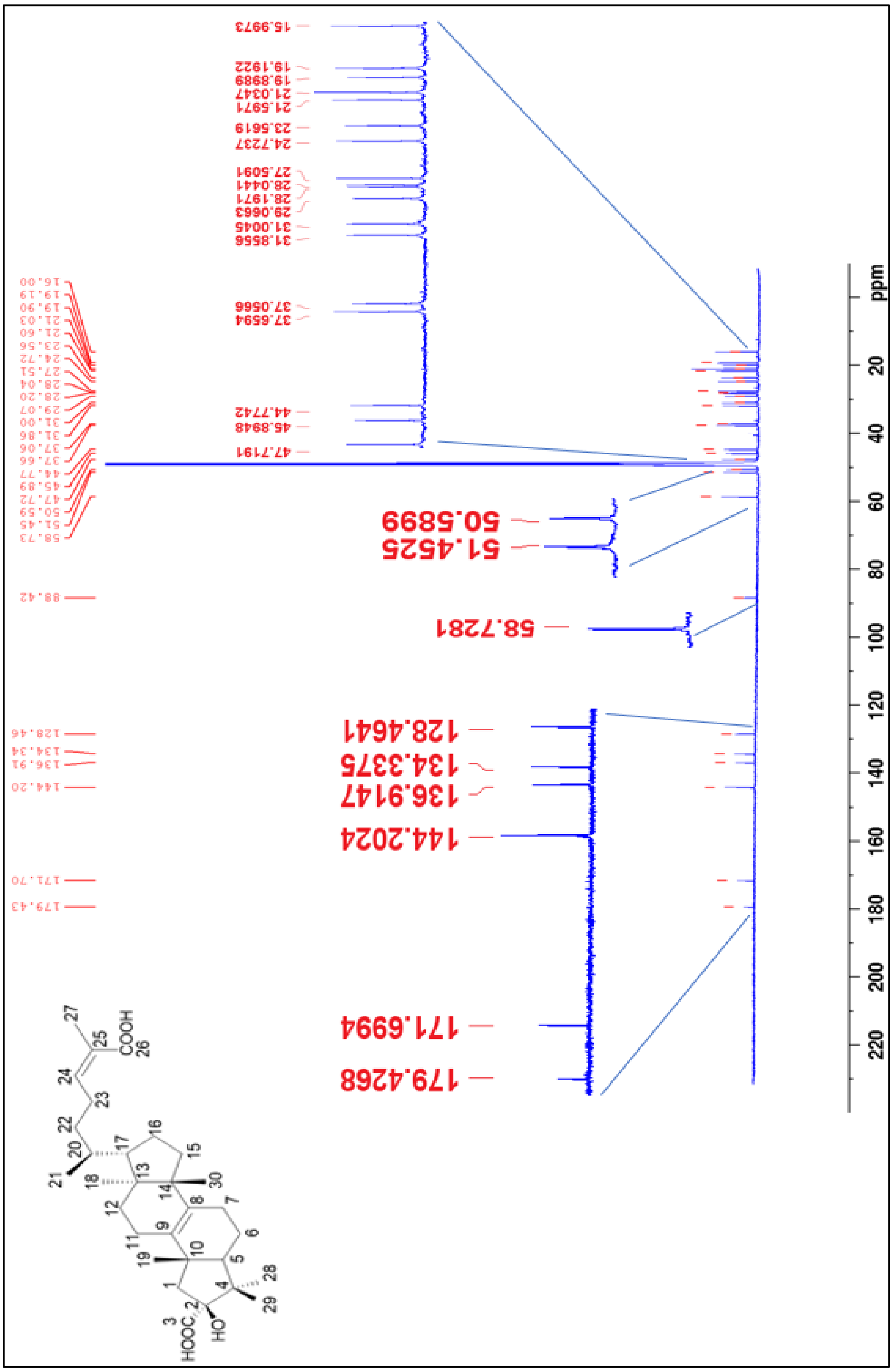

Figure S35: ^{13}C (151 MHz, MeOD) spectrum of **3**.

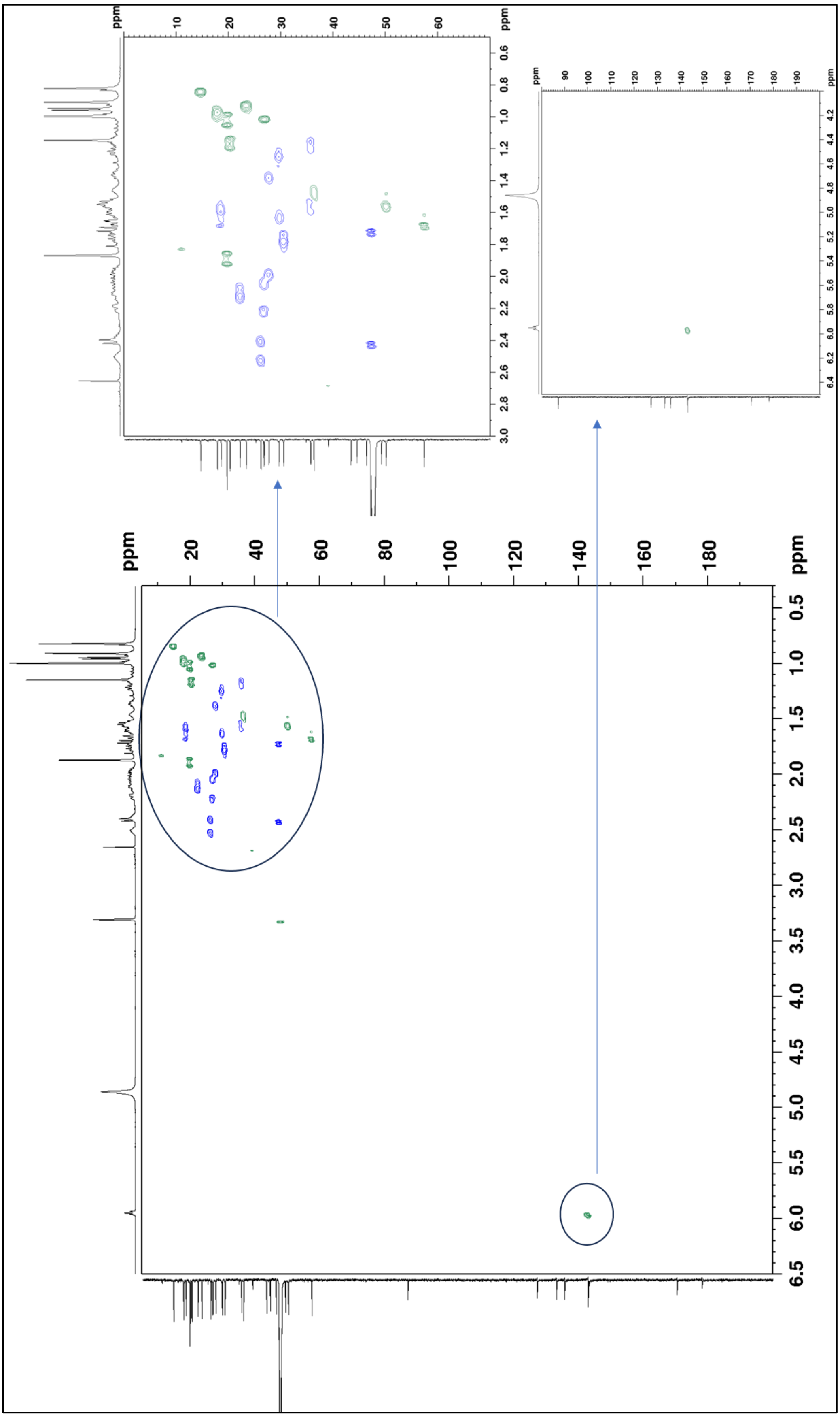

Figure S36: HSQC-DEPT (600 MHz, MeOD) spectrum of **3**.

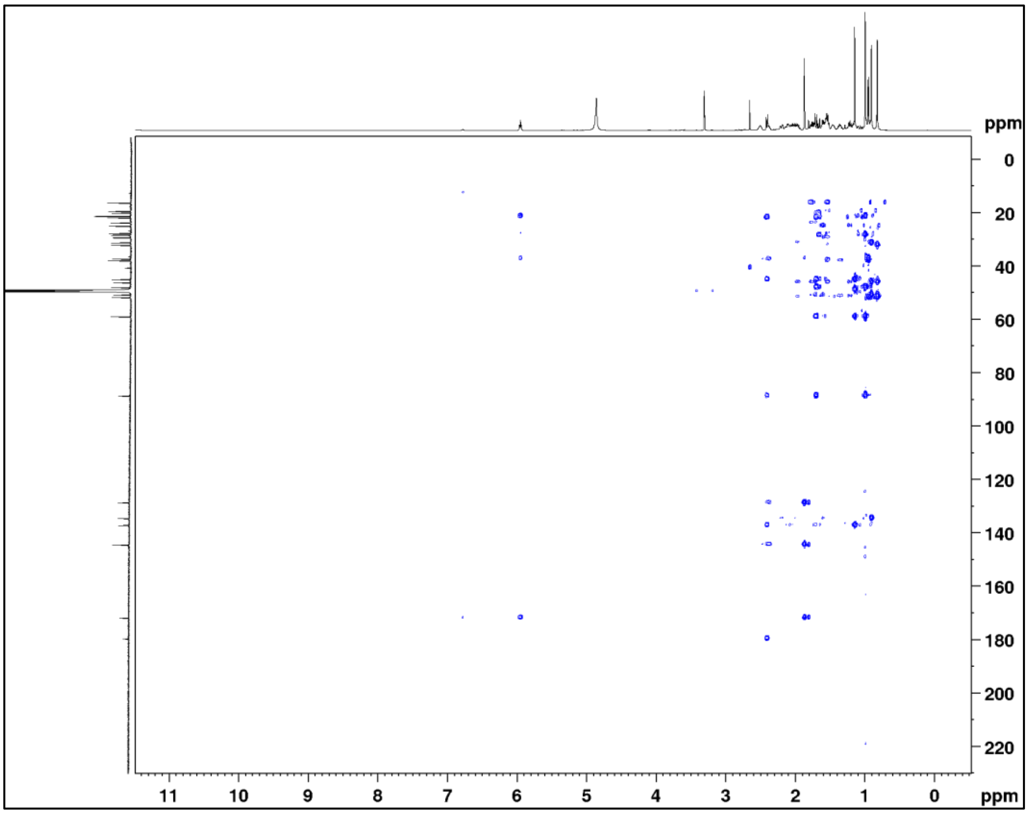

Figure S37: HMBC (600 MHz, MeOD), spectrum of **3**.

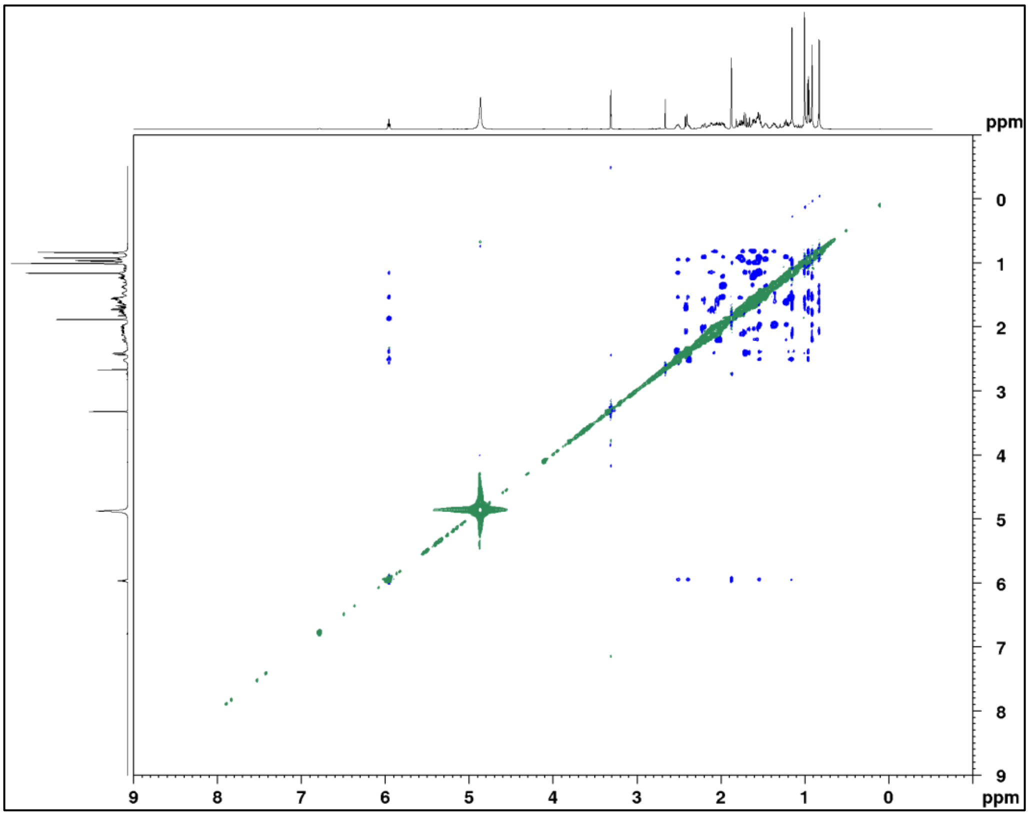

Figure S38: NOESY (600 MHz, MeOD) spectrum of **3**.
